# Proteome Based Construction of the Lymphocyte Function-Associated Antigen 1 (LFA-1) Interactome in Human Dendritic Cells

**DOI:** 10.1371/journal.pone.0149637

**Published:** 2016-02-18

**Authors:** Christina Eich, Edwin Lasonder, Luis J. Cruz, Inge Reinieren-Beeren, Alessandra Cambi, Carl G. Figdor, Sonja I. Buschow

**Affiliations:** 1 Department of Tumor Immunology, Radboud Institute for Molecular Life Sciences, Radboud University Medical Centre, Nijmegen, The Netherlands; 2 CMBI, Radboud Institute for Molecular Life Sciences, Radboud University Nijmegen Medical Centre, Nijmegen, The Netherlands; 3 Nanomedicine and Molecular Imaging, Department of Radiology, Leiden University Medical Center, Leiden, The Netherlands; Istituto Superiore di Sanità, ITALY

## Abstract

The β2-integrin lymphocyte function-associated antigen 1 (LFA-1) plays an important role in the migration, adhesion and intercellular communication of dendritic cells (DCs). During the differentiation of human DCs from monocyte precursors, LFA-1 ligand binding capacity is completely lost, even though its expression levels were remained constant. Yet LFA-1-mediated adhesive capacity on DCs can be regained by exposing DCs to the chemokine CCL21, suggesting a high degree of regulation of LFA-1 activity during the course of DC differentiation. The molecular mechanisms underlying this regulation of LFA-1 function in DCs, however, remain elusive. To get more insight we attempted to identify specific LFA-1 binding partners that may play a role in regulating LFA-1 activity in DCs. We used highly sensitive label free quantitative mass-spectrometry to identify proteins co-immunoprecipitated (co-IP) with LFA-1 from *ex vivo* generated DCs. Among the potential binding partners we identified not only established components of integrin signalling pathways and cytoskeletal proteins, but also several novel LFA-1 binding partners including CD13, galectin-3, thrombospondin-1 and CD44. Further comparison to the LFA-1 interaction partners in monocytes indicated that DC differentiation was accompanied by an overall increase in LFA-1 associated proteins, in particular cytoskeletal, signalling and plasma membrane (PM) proteins. The here presented LFA-1 interactome composed of 78 proteins thus represents a valuable resource of potential regulators of LFA-1 function during the DC lifecycle.

## Introduction

Integrins form a family of heterodimeric transmembrane receptors that regulate cell–adhesion and cell-extracellular matrix interactions. *Lymphocyte function-associated antigen 1* (LFA-1, αLβ2) is a leukocyte specific integrin that mediates firm arrest of leukocytes on the endothelium during migration and establishes cell-cell contacts such as the immunological synapse between DCs and T cells [1]. At the molecular level, transient modifications of adhesion are accomplished by conformational changes of LFA-1 from a bent down inactive to an upright active conformation with an open headpiece, leading to low- and high-affinity for its major ligand ICAM-1 respectively [2]. These conformational changes are induced by binding of the cytoskeletal protein talin as the terminal step in a process termed “inside-out” signalling where intracellular signalling events lead to changes in integrin activity and alter cell adhesion [3]. In the context of cell-adhesion, integrins in turn deliver “outside-in” signals upon ligand binding that control cell proliferation, survival, gene induction, differentiation and cell motility. To achieve this, integrins recruit molecular complexes consisting of numerous cytoskeletal and signalling molecules [4].

Several studies have addressed the interaction partners of integrins in ligand-induced adhesion complexes [5–9] and in different modes of cell adhesion [10]. So far, over 190 proteins from different cellular systems have been described to constitute the focal adhesion network [7,11], which led to the identification of key regulatory proteins acting during the adhesion to different ligands [6]. However, little is currently known about the steady-state organization of integrins within the plasma membrane (PM) and the molecules involved in regulating their activity prior to ligand encounter. In an attempt to describe a leukocyte-specific “pro-adhesive” integrin network, Laudanna and colleagues summarized 64 proteins that are involved in integrin regulation prior to ligand binding, which had been derived from different experimental systems and cell types [12]. How exactly the molecular assembly and interaction partners prior to ligand encounter vary between integrins, cell types and differentiation states, and how molecular pre-assemblies relate to the spatiotemporal regulation of integrin function, remains elusive.

We have previously shown that during *in vitro* differentiation of primary human monocytes towards immature monocyte-derived DCs (imDCs), LFA-1 remains expressed at similar levels, but loses its upright/functional form. In addition, DC LFA-1 is no longer organized in defined PM nanoclusters of ~100nm in size but rather has a random distribution [13,14]. As recently shown by our group, DC LFA-1 however could be reactivated by the chemokine CCL21 [15] indicating that LFA-1 adhesive properties are subject to a high level of regulation during DC differentiation and exposure to external stimuli. In this respect, others have shown that pre-organized signalling platforms may facilitate ligand encounter and/ or promote a rapid transmission and response to intracellular signalling events [16–18], which could play a role in the regulation of LFA-1 in DCs. To identify proteins associated with LFA-1 on *in vitro* cultured DCs that may constitute such signalling platforms we here used selective immuno-precipitation (IP) of LFA-1 followed by highly sensitive label free quantitative mass-spectrometry (MS) analysis. Our results describe a unique signature of LFA-1 interacting proteins in DCs, containing established and potentially novel LFA-1 binding partners. Only part of this DC derived LFA-1 interactome was also connected to LFA-1 in monocytes. These proteins may have important implications for the adhesive properties of LFA-1 in DCs and in other cell types. Based on these identified interaction partners we reconstructed a DC-specific LFA-1 Protein-Protein Interaction (PPI) network involving 78 proteins that represents a great resource for future studies on the molecular regulation of LFA-1 function.

## Materials and Methods

### Cells

Monocytes were isolated from buffy coats (Sanquin) obtained from healthy volunteers after written informed consent and according to institutional guidelines, verified by the local institutional review board (Commissie mensgebonden onderzoek (CMO)). Peripheral blood mononuclear cells were purified from buffy coats using Ficoll density centrifugation. Monocytes were isolated by plate adherence. DCs were obtained by culturing adherent monocytes in the presence of interleukin-4 (300U/ml) and granulocyte macrophage colony-stimulating factor (450U/ml) (both from Strathmann, Hamburg Germany). MoDCs were cultured for 6 days to obtain imDCs.

### Immunoprecipitation

Monocytes and imDCs were thoroughly washed with PBS and subsequently lysed directly in the flask with lysis buffer (20mM Tris pH 8, 150mM NaCl, 1mM CaCl_2_, 1mM MgCl_2_) containing either 1% Brij 97 (Brij buffer) or 1% Nonidet P-40 + 0.5% sodium deoxycholate + 0.1% SDS (RIPA buffer), in the presence of protease (Complete, Roche) and phosphatase inhibitors (Phosphostop Roche). After 15 min lysis on ice, cell lysates were cleared by centrifugation at 14000g for 15 min. First, cell lysates were precleared with bovine serum albumin-coated protein G sepharose beads (GE Healthcare), followed by empty protein G sepharose beads in the presence of 1/1000 volume heat inactivated goat serum, and finally by preclearing with mouse IgG1 coated (2μg) protein G sepharose beads. Each step was performed at 4°C for 1 hour. Proteins were then immunoprecipitated by adding protein G sepharose beads coated with 5μg of specific anti-CD11a antibody (SPV-L7 [19]) or 5μg unspecific mouse IgG1 isotype antibody to 400μl lysate. After 2 h incubation under constant agitation at 4°C, beads were washed five times in Brij 97 or RIPA lysis buffer. The immunoprecipitates were separated by SDS-PAGE under reducing conditions. Proteins in the gel were visualized with colloidal coomassie staining (Invitrogen).

### Western blotting

Proteins were separated by SDS-PAGE and transferred to PVDF membranes (Millipore, Bedford, MA). Subsequently, proteins were detected by immunoblotting. After labelling, Western blots were scanned by the Odyssey imager (LI-COR Biosciences).

### Microscopy

For co-capping experiments, day 6 monocyte-derived imDCs were double-stained at 4°C with 5μg/ml anti–LFA-1 mAb (clone NKI-L15, mIgG2a or clone TS2/4, mIgG1) and 5μg/ml mAbs against Galectin-3 (clone M3/38, ratIgG2a), CD44 (clone G44-26, mIgG2b), CD71 (clone b3/25, mIgG1) or CD13 (clone WM15, mIgG1). Isotype-specific controls were always included. Secondary staining was performed with Alexa 488–conjugated goat anti–mouse IgG2a or IgG2b and Alexa 647–conjugated goat anti–mouse IgG1. Patching was induced by incubation at 15°C for 1 h, followed by fixation with 1% paraformaldehyde. Cells were mounted onto poly-l-lysine–coated glass coverslips. Fixed cells were imaged on an FV1000 confocal laser scanning microscope (Olympus, Tokyo, Japan) equipped with argon (488 nm) 559- and 635-nm diode lasers using a PlanApochromatic 60×/1.35 numerical aperture (NA) oil immersion objective.

Signals were collected sequentially to avoid bleed through. The Pearson correlation coefficient was calculated by applying the Image J (http://rsb.info.nih.gov/ij/,) plug-in JACoP [20] and reflects the amount of LFA-1 co-localizing with Galectin-3, CD44, CD13 or CD71 separately for each cell.

### Sample preparation for LC-MS/MS experiments

Protein samples were loaded on a 8% SDS PAGE gel, followed by a short electrophoresis period of 5 minutes, stained with colloidal Coommassie blue and divided into a single gel slice per sample. Gel slices were treated with dithiothreitol (DTT) and iodoacetamide and digested by trypsin [21]. Digested samples were acidified to a final concentration of 0.1% trifluoroacetic acid and purified by self-made and extremely economical stop-and-go-extraction tips (STAGE) tips [22].

### Liquid chromatography tandem mass spectrometry

Peptide sequencing experiments were performed by LC-MS/MS using a nano HPLC Agilent 1100 LC system connected to a 7-Tesla linear ion trap ion cyclotron resonance Fourier transform (LTQ-FT Ultra) mass spectrometer (Thermo Fisher, Bremen, Germany) and measured as described previously [21]. Peptides were separated on 15 cm 100 μm ID PicoTip columns (New Objective, Woburn, USA) packed with 3 μm Reprosil C18 beads (Dr. Maisch GmbH, Ammerbuch, Germany) using a 120 min gradient from 12% buffer B to 40% buffer B (buffer B contains 80% acetonitrile in 0.5% acetic acid) with a flow-rate of 300 nl/min The mass spectrometer was operated with a spray voltage of 2.2 kV, and data was acquired in a data-dependent mode with full-scan MS spectra of intact peptides (m/z 350–1500) with an automated gain control accumulation target value of 1,000,0000 ions in the Fourier transform ion cyclotron resonance cell with a resolution of 50,000. The four most abundant ions were sequentially isolated and fragmented in the linear ion trap by applying collisionally induced dissociation using an accumulation target value of 10,000, a capillary temperature of 100°C, and a normalized collision energy of 27%. A dynamic exclusion of ions previously sequenced within 180 s was applied. All unassigned charge states and singly charges ions were excluded from sequencing. A minimum of 200 counts was required for MS2 selection. Maximum injection times were set at 500 ms and 400 ms respectively for FT MS and IT MS/MS measurements.

### Protein identification and quantification

Raw spectrum files were processed for identification by the Andromeda search engine [23] and quantification according to the iBAQ method [24] with MaxQuant version1.2.0.18 [25]. (http://maxquant.org/). Peak lists were generated using the default setting that extracted the top 6 MS MS peaks per 100 Da. Proteins were identified by searching peak lists with Andromeda against the human International Protein Index (IPI) database version 3.68 (ftp://ftp.ebi.ac.uk/pub/databases/IPI/) supplemented with frequently observed contaminants using the target decoy approach. Search parameters for protein identification specified a mass tolerance of 7ppm for the parental peptide and 0.5 Da for fragmentation spectra and a trypsin specificity allowing up to 3 miscleaved sites. Carbamidomethylation of cysteines was specified as a fixed modification, and oxidation of methionine, deamidation of glutamine and asparagine, and acetylation of the protein N-terminus were set as variable modifications. The required minimal peptide length was set at 6 amino acids. Peptide validation by establishing false discovery rates (FDR) was performed by MaxQuant as described [25]. We accepted peptides (charge state >1, nr variable modifications <4) with peptide FDR of 1% and proteins with a protein PEP<0.002 and protein FDR of 1%.

Proteins were quantified by label free quantification using the intensity- based absolute quantification (iBAQ) [24]. Protein intensities were calculated by MaxQuant as the normalised sum of all identified peptide intensities s. Protein intensities were determined in duplicate measurements, then normalised by median iBAQ value and finally averaged. The criteria for protein selection were the following: for stringent IP conditions we selected proteins that were at least 2.5 fold enriched over the isotype control (based on the normalized IBAQ value), or that were specifically detected in the LFA-1 IP by at least 3 unique peptides. In mild IP conditions we included proteins that were 2.5 fold enriched over the isotype control, or, if LFA-1 IP specific, detected with at least 2 peptides.

### Protein protein interaction network analysis

The STRING (STRING v9.1) database (http://string-db.org/) [26] was used to retrieve high confidence (0.6), direct protein interactions (based on experimental evidence) within the lists of putative LFA-1 binding partners in monocytes and DCs, separately. The final PPI network layout was redrawn by the authors, based on the graphical PPI network generated in STRING.

### The Ingenuity pathways analysis (IPA)

The Ingenuity pathways analysis (IPA) software (Ingenuity Systems, Redwood City, CA) is a bioinformatics tool that puts experimental data in relation to published research by identifying relationships, mechanisms, functions, and pathways of relevance. The LFA-1 binding candidates in monocytes and DC were analysed for the top 5 most enriched IPA pathways against a background of all human proteins by right tailed Fisher Exact Tests in a core analysis calculating the likelihood that this is due to random chance.

### Antibodies

The following primary antibodies were used for Western blotting and confocal analysis: mouse anti-human LFA-1 (clone NKI-L15 and TS2/4, hybridoma), rabbit anti-human LFA-1 (EP1285Y, Abcam), rat-anti Galectin-3 (clone M3/38, Biolegend), mouse anti-human CD13 (clone WM15, biolegend), mouse anti-human CD44 (clone G44-26, BD biosciences), mouse anti-human Talin-1 (clone 8d4, Sigma), mouse anti- Actin (clone AC-40, Sigma), rabbit anti-human thrombospondin-1 (Abcam), mouse anti-human CD71 (clone b3/25, hybridoma). IRDye conjugated secondary antibodies for Western blotting were obtained from LI-COR Biosciences, and Alexa-647 conjugated secondary antibodies for flow cytometry and confocal analysis were from Invitrogen. For LFA-1 immunoprecipitation studies, an antibody directed specifically against the αL chain was used (clone SPV-L7).

## Results

### Isolation of LFA-1 associated proteins in DCs

**Fig 1:**
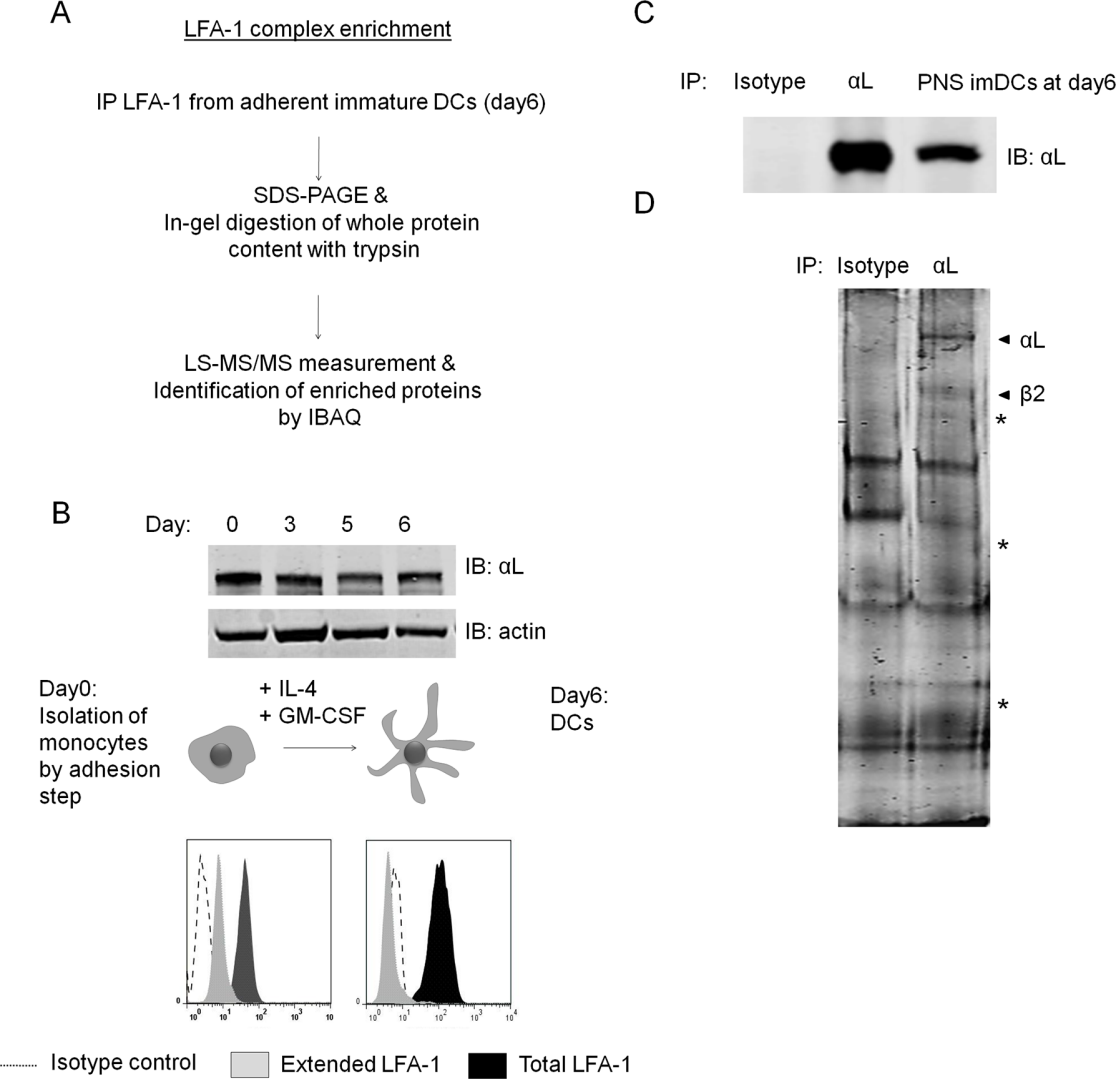
Enrichment and identification of LFA-1 complexes. (A) Experimental flow chart for enrichment and identification of LFA-1 complexes in imDCs. Adherent imDCs from 3 healthy donors were lysed, followed by enrichment of LFA-1 by IP and separation by SDS-PAGE. The protein content was visualized by colloidal coomassie labeling, excised in one piece and subjected to MS analysis and protein identification. (B) Top: Immunolabeling of LFA-1 in cell lysates of equal amounts of monocytes (day0) and imDCs on day 3,5 and 6 of DC differentiation. Loading control: actin. Bottom: Expression levels of total (TS2/4) and active/extended (NKI-L16) cell surface LFA-1 assessed by flow cytometry. (Isotype control, dotted line; extended LFA-1, grey; total LFA-1, black). (C) Immunolabeling of immunoprecipitated LFA-1 from the post nuclear supernatant (PNS) of day 6 imDCs. (D) Colloidal coomassie staining of SDS-PAGE gels from immunoprecipiated LFA-1 in DCs. LFA-1 was enriched using mAb (clone SPV-L7) directed against αL. mIgG1 coated beads were included as control IP. LFA-1 subunits and potential interaction partners are indicated by arrow heads. Protein bands specifically identified in the LFA-1 IP are indicated by asterisk.

To investigate which proteins are associated with LFA-1 in DCs prior to ligand binding, we performed IPs of LFA-1 from primary human *in vitro* derived DCs and analyzed precipitated proteins by quantitative nano liquid chromatography tandem mass spectrometry (LC-MS/MS) (Fig 1A). Western blotting (WB) and FACS analysis confirmed our previous finding that the LFA-1 protein levels remained steady during differentiation of monocytes towards DC (Fig 1B), while the cell surface expression of the extended/functional conformation of LFA-1 was lost in DCs [13] (Fig 1B). LFA-1 complexes were efficiently purified from DCs by beads coated with a specific antibody directed at the αL chain of LFA-1 (Fig 1C). To retrieve only proteins strongly interacting with LFA-1 and to obtain a high confidence of the interaction, cell lysis and IP were performed in the presence of a stringent detergent on DCs obtained from 3 different donors. As expected based on our WB results, colloidal Coomassie staining showed a clear presence of protein at the expected molecular weight of the integrin αL- and β-chain. In addition, the Coomassie stained gel already revealed several other proteins specifically present in the LFA-1 precipitate of DCs that were not precipitated with an isotype-matched control antibody (Fig 1D). Both IP samples were subsequently subjected to LC-MS/MS and proteins were identified and quantified by MaxQuant applying the label free iBAQ method [24]. Both the larger number of peptides derived from αL and β2 identified by mass spectrometry as well as the approximately 10 fold higher iBAQ values for both subunits, confirmed enrichment of LFA-1 in the LFA-1 targeting IP with respect the isotype control-IP, as expected from the Western blot data (Table 1; Fig 1).

**Table 1:** Protein binding partners identified in stringent IP conditions in DCs.

	Protein AcNR	HGNC symbol	Protein Name (IPA)	normalized IBAQ control IP	normalized IBAQ LFA-1 IP	max peptides control	max peptides LFA-1 IP	fold change (LFA-1 IP/Control IP)
1	IPI00221224	ANPEP	alanyl (membrane) aminopeptidase	nd	18.25	0	15	LFA-1 IP specific
2	IPI00221226	ANXA6	annexin A6	nd	4.30	0	11	LFA-1 IP specific
3	IPI00333541	FLNA	filamin A, alpha	nd	0.99	0	11	LFA-1 IP specific
4	IPI00867509	CORO1C	coronin, actin binding protein, 1C	nd	9.81	0	11	LFA-1 IP specific
5	IPI00294739	SAMHD1	SAM domain and HD domain 1	nd	4.55	0	9	LFA-1 IP specific
6	IPI00024067	CLTC	clathrin, heavy chain (Hc)	nd	0.63	0	8	LFA-1 IP specific
7	IPI00171903	HNRNPM	heterogeneous nuclear ribonucleoprotein M	nd	2.01	0	8	LFA-1 IP specific
8	IPI00793199	ANXA4	annexin A4	nd	6.08	0	7	LFA-1 IP specific
9	IPI00477313	HNRNPC	heterogeneous nuclear ribonucleoprotein C (C1/C2)	nd	7.75	0	5	LFA-1 IP specific
10	IPI00299719	TCIRG1	T-cell, immune regulator 1, ATPase, H+ transporting, lysosomal V0 subunit A3	nd	0.90	0	4	LFA-1 IP specific
11	IPI00304171	H2AFY	H2A histone family, member Y	nd	2.04	0	4	LFA-1 IP specific
12	IPI00873622	WDR1	WD repeat domain 1	nd	0.93	0	4	LFA-1 IP specific
13	IPI00909703	ANXA11	annexin A11	nd	7.09	0	4	LFA-1 IP specific
14	IPI00008530	RPLP0	ribosomal protein, large, P0	nd	1.08	0	3	LFA-1 IP specific
15	IPI00019912	HSD17B4	hydroxysteroid (17-beta) dehydrogenase 4	nd	0.52	0	3	LFA-1 IP specific
16	IPI00027438	FLOT1	flotillin 1	nd	0.43	0	3	LFA-1 IP specific
17	IPI00220834	XRCC5	X-ray repair complementing defective repair in Chinese hamster cells 5 (double-strand-break rejoining)	nd	0.71	0	3	LFA-1 IP specific
18	IPI00295857	COPA	coatomer protein complex, subunit alpha	nd	0.34	0	3	LFA-1 IP specific
19	IPI00298994	TLN1	talin 1	nd	0.06	0	3	LFA-1 IP specific
20	IPI00305064	CD44	CD44 molecule (Indian blood group)	nd	1.46	0	3	LFA-1 IP specific
21	IPI00465431	LGALS3	lectin, galactoside-binding, soluble, 3	nd	13.10	0	3	LFA-1 IP specific
22	IPI00855785	FN1	fibronectin 1	nd	0.34	0	3	LFA-1 IP specific
23	IPI00902560	VDAC2	voltage-dependent anion channel 2	nd	1.29	0	3	LFA-1 IP specific
24	IPI00939163	HSPH1	heat shock 105kDa/110kDa protein 1	nd	0.49	0	3	LFA-1 IP specific
25	IPI00418169	ANXA2	annexin A2	4.46	993.57	8	22	223.02
26	IPI00010477	LGALS9	lectin, galactoside-binding, soluble, 9	0.56	34.79	1	3	61.98
27	IPI00021812	AHNAK	AHNAK nucleoprotein	0.12	7.44	1	71	61.70
28	IPI00216691	PFN1	profilin 1	0.40	14.58	1	5	36.02
29	IPI00453473	PPIA	peptidylprolyl isomerase A (cyclophilin A)	2.51	84.31	2	3	33.63
30	IPI00646240	HIST2H2BF	histone cluster 2, H2bf	0.62	18.80	1	4	30.44
31	IPI00294578	TGM2	transglutaminase 2	0.68	14.51	5	11	21.23
32	IPI00005159	ACTR2	ARP2 actin-related protein 2 homolog (yeast)	2.83	59.03	4	4	20.85
33	IPI00169383	PGK1	phosphoglycerate kinase 1	0.08	1.42	2	5	17.37
34	IPI00011644	PTPRE	protein tyrosine phosphatase, receptor type, E	0.08	1.24	1	6	15.58
35	IPI00029741	ITGB5	integrin, beta 5	1.09	16.59	1	1	15.25
36	IPI00291792	ITGB2	integrin, beta 2	4.62	62.62	10	20	13.54
37	IPI00013163	MNDA	myeloid cell nuclear differentiation antigen	0.07	0.80	2	3	11.20
38	IPI00003918	RPL4	ribosomal protein L4	0.25	2.76	1	3	10.92
39	IPI00016342	RAB7A	RAB7A, member RAS oncogene family	0.27	2.90	1	3	10.75
40	IPI00926935	GNAI2	guanine nucleotide binding protein (G protein), alpha inhibiting activity polypeptide 2	0.31	3.24	1	4	10.37
41	IPI00941721	PTPN6	protein tyrosine phosphatase, non-receptor type 6	0.07	0.67	1	3	9.83
42	IPI00025380	ITGAL	integrin, alpha L (antigen CD11A (p180), lymphocyte function-associated antigen 1; alpha polypeptide)	6.34	60.23	20	28	9.50
43	IPI00291764	SLC25A6	solute carrier family 25 (mitochondrial carrier; adenine nucleotide translocator), member 6	1.51	13.85	1	3	9.20
44	IPI00555744	RPL14	ribosomal protein L14	0.23	1.88	1	1	8.26
45	IPI00180675	TUBA1A	tubulin, alpha 1a	0.22	1.78	1	2	8.12
46	IPI00883857	HNRNPU	heterogeneous nuclear ribonucleoprotein U (scaffold attachment factor A)	0.21	1.67	1	5	8.00
47	IPI00215914	ARF1	ADP-ribosylation factor 1	1.63	12.69	1	4	7.77
48	IPI00010471	LCP1	lymphocyte cytosolic protein 1 (L-plastin)	1.07	8.12	7	13	7.58
49	IPI00005969	CAPZA1	capping protein (actin filament) muscle Z-line, alpha 1	0.35	2.50	1	2	7.24
50	IPI00009342	IQGAP1	IQ motif containing GTPase activating protein 1	0.06	0.46	2	5	7.20
51	IPI00028091	ACTR3	ARP3 actin-related protein 3 homolog (yeast)	1.37	9.05	5	6	6.61
52	IPI00550069	RNH1	ribonuclease/angiogenin inhibitor 1	0.28	1.86	1	4	6.54
53	IPI00925520	PFKL	phosphofructokinase, liver	0.07	0.44	1	2	5.91
54	IPI00010415	ACOT7	acyl-CoA thioesterase 7	0.10	0.55	1	3	5.40
55	IPI00376798	RPL11	ribosomal protein L11	0.66	3.40	1	1	5.13
56	IPI00215719	RPL18	ribosomal protein L18	0.72	3.65	1	1	5.06
57	IPI00414676	HSP90AB1	heat shock protein 90kDa alpha (cytosolic), class B member 1	1.96	9.85	5	9	5.04
58	IPI00179330	RPS27A	ribosomal protein S27a	3.20	15.66	2	2	4.89
59	IPI00930688	TUBA1B	tubulin, alpha 1b	4.66	21.68	5	10	4.66
60	IPI00013508	ACTN1	actinin, alpha 1	2.58	11.80	16	24	4.57
61	IPI00396378	HNRNPA2B1	heterogeneous nuclear ribonucleoprotein A2/B1	0.69	2.71	2	3	3.94
62	IPI00219910	BLVRB	Biliverdin Reductase B (Flavin Reductase (NADPH))	0.54	2.11	1	1	3.92
63	IPI00871870	ARPC3	actin related protein 2/3 complex, subunit 3, 21kDa	1.14	4.41	1	1	3.87
64	IPI00641829	DDX39B	DEAD (Asp-Glu-Ala-Asp) box polypeptide 39B	0.84	3.06	3	3	3.65
65	IPI00645452	TUBB	tubulin, beta class I	7.69	26.75	12	12	3.48
66	IPI00456887	HNRNPUL2	heterogeneous nuclear ribonucleoprotein U-like 2	0.15	0.50	1	1	3.43
67	IPI00792352	LIPA	lipase A, lysosomal acid, cholesterol esterase	0.87	2.99	1	2	3.41
68	IPI00011253	RPS3	ribosomal protein S3	0.45	1.50	2	4	3.36
69	IPI00303476	ATP5B	ATP synthase, H+ transporting, mitochondrial F1 complex, beta polypeptide	1.08	3.62	4	6	3.35
70	IPI00027230	HSP90B1	heat shock protein 90kDa beta (Grp94), member 1	0.33	1.07	1	6	3.26
71	IPI00016610	PCBP1	poly(rC) binding protein 1	1.17	3.78	3	3	3.22
72	IPI00604523	MYL12A	myosin, light chain 12A, regulatory, non-sarcomeric	33.54	106.22	7	7	3.17
73	IPI00397834	FERMT3	fermitin family member 3	0.99	2.98	4	8	3.00
74	IPI00008964	RAB1B	RAB1B, member RAS oncogene family	0.12	0.35	1	1	2.98
75	IPI00005161	ARPC2	actin related protein 2/3 complex, subunit 2, 34kDa	2.20	6.46	5	6	2.93
76	IPI00019884	ACTN2	actinin, alpha 2	0.07	0.21	1	1	2.91
77	IPI00221221	ALOX15	arachidonate 15-lipoxygenase	1.13	3.27	7	9	2.90
78	IPI00295741	CTSB	cathepsin B	0.26	0.73	1	1	2.82
79	IPI00294158	BLVRA	biliverdin reductase A	0.25	0.70	1	1	2.76
80	IPI00027107	TUFM	Tu translation elongation factor, mitochondrial	0.18	0.48	1	1	2.67
81	IPI00926581	MYH14	myosin, heavy chain 14, non-muscle	23.43	61.93	3	2	2.64
82	IPI00215918	ARF4	ADP-ribosylation factor 4	0.29	0.75	1	1	2.59
83	IPI00025491	EIF4A1	eukaryotic translation initiation factor 4A1	1.37	3.48	2	4	2.53

The criteria for protein selection were the following: proteins were at least 2.5 fold enriched over the isotype-type matched control IP (based on the normalized IBAQ value), or were specifically detected in the LFA-1 IP with a minimum of 3 unique peptides. Proteins were mapped to HGNC and International Protein Index (IPI) identifiers. nd = not detected.

### Identification of LFA-1 associated proteins in DCs

To derive candidate binding partners of LFA-1 we took into account only proteins that were either detected specifically in the LFA-1 IP with a minimum of 3 peptides and were absent in the control IP, or that were at least 2.5 fold enriched in the LFA-1 IP (based on normalized IBAQ value) over the control IP. Applying those criteria, a total of 94 entries were scored as potential interaction partners of LFA-1 in DCs (Table 1). These entries corresponded to the specific international protein index (IPI) and could be mapped to 83 HUGO Gene Nomenclature Committee (HGNC) annotated proteins. Among the interaction partners we found the established integrin binding partners, kindlin-3 (FERMT3) and talin-1 (TLN1), as well as many other proteins that are known to be involved in integrin signaling and cell adhesion and that may thus play a role in the regulation of LFA-1 function (Table 1).

**Fig 2:**
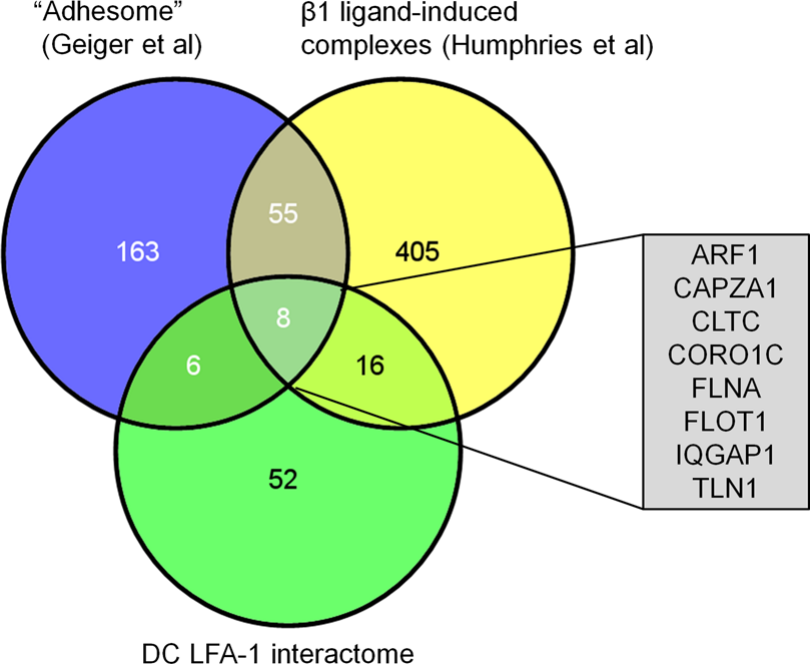
Comparison of LFA-1 binding partners in DCs with published ligand-induced integrin complexes. Venn diagrams of proteins identified in this study (green), proteins summarized in the focal adhesion adhesome by Geiger et al [11] (blue) and proteins identified in experimental ligand induced integrin complexes by Humphries et al [6] (yellow). Numbers of identified proteins, as well as gene symbols of commonly identified proteins by Geiger et al, Humphries et al and this study are indicated.

When comparing these interactions partners to components of published ligand-induced integrin-complexes [6,7,11], we found an overlap of 14 proteins with the adhesome and of 24 proteins with a proteomic analysis of β1integrin-associated complexes [6]. In total, 32 of the here identified proteins (38.5%) overlapped with published integrin interactomes (Fig 2). This overlap is quite high, considering that the highlighted studies focused on other integrins and, importantly, were performed in the presence of ligand. In contrast, we studied proteins interacting with LFA-1 at steady-state LFA-1 prior to ligand binding. This suggests that the overlapping proteins may represent more general and unconditional integrin interaction partners. The eight proteins shared among all data sets were ADP-ribosylation factor 1 (ARF1), capping proteins (actin filament) muscle Z-line alpha1 (CAPZA1), Clathrin heavy chain 1 (CLTC), coronin1C (CORO1C), filamin A (FLNA), flotillin 1 (FLOT1), Ras GTPase-activating-like protein IQGAP1 (IQGAP1) and talin-1 (Fig 2). Uniquely shared with the two datasets were mainly cytoskeletal proteins and, in case of the adhesome, also some ribosomal proteins which most likely represent a remaining pollution of these highly abundant proteins inevitably present in unbiased proteome analysis.

To further highlight the enrichment of proteins relevant for integrin function in our dataset and to understand which molecular pathways the identified LFA-1 interaction partners are assigned to, we performed ingenuity pathway analysis (IPA). First, gene IDs of LFA-1 binding candidates were mapped to the Ingenuity’s knowledge database containing predefined canonical pathways. The LFA-1 interaction partners in DCs were significantly overrepresented in the canonical pathway “integrin signaling” (p = 3.17E-12), confirming selective precipitation of proteins involved in integrin function (Table 2). Also overrepresented were the pathways “Remodeling of Epithelial Adherens Junctions” (p = 5.52E-15), “Actin Cytoskeleton Signaling” (p = 7.85E-12) and “Epithelial Adherens Junction Signaling” (p = 3.01E-11), (Table 2), which also clearly relate to integrin activity. We then evaluated the coverage of these signaling pathways by defining the fraction of proteins in the data to the total size of the pathway. The coverage ranged from 6–16%, with the highest coverage in the pathway “Remodeling of Epithelial Adherens Junctions” (Table 2).

**Table 2:** Ingenuity Pathway Analysis: Top Canonical Pathways in DCs (stringent IP conditions).

	Name	Fisher Exact Test p value	Pathway coverage
**1**	**Remodeling of Epithelial Adherens Junctions**	**5,52E-15**	**16.2%**
**2**	**Integrin Signaling**	**3,17E-12**	**6.4%**
**3**	**Actin Cytoskeleton Signaling**	**7,85E-12**	**6%**
**4**	**Epithelial Adherens Junction Signaling**	**3,02E-11**	**7.5%**
**5**	**Paxillin Signaling**	**2,69E-07**	**6.9%**

### Validation of LFA-1 binding partners in DCs

**Fig 3:**
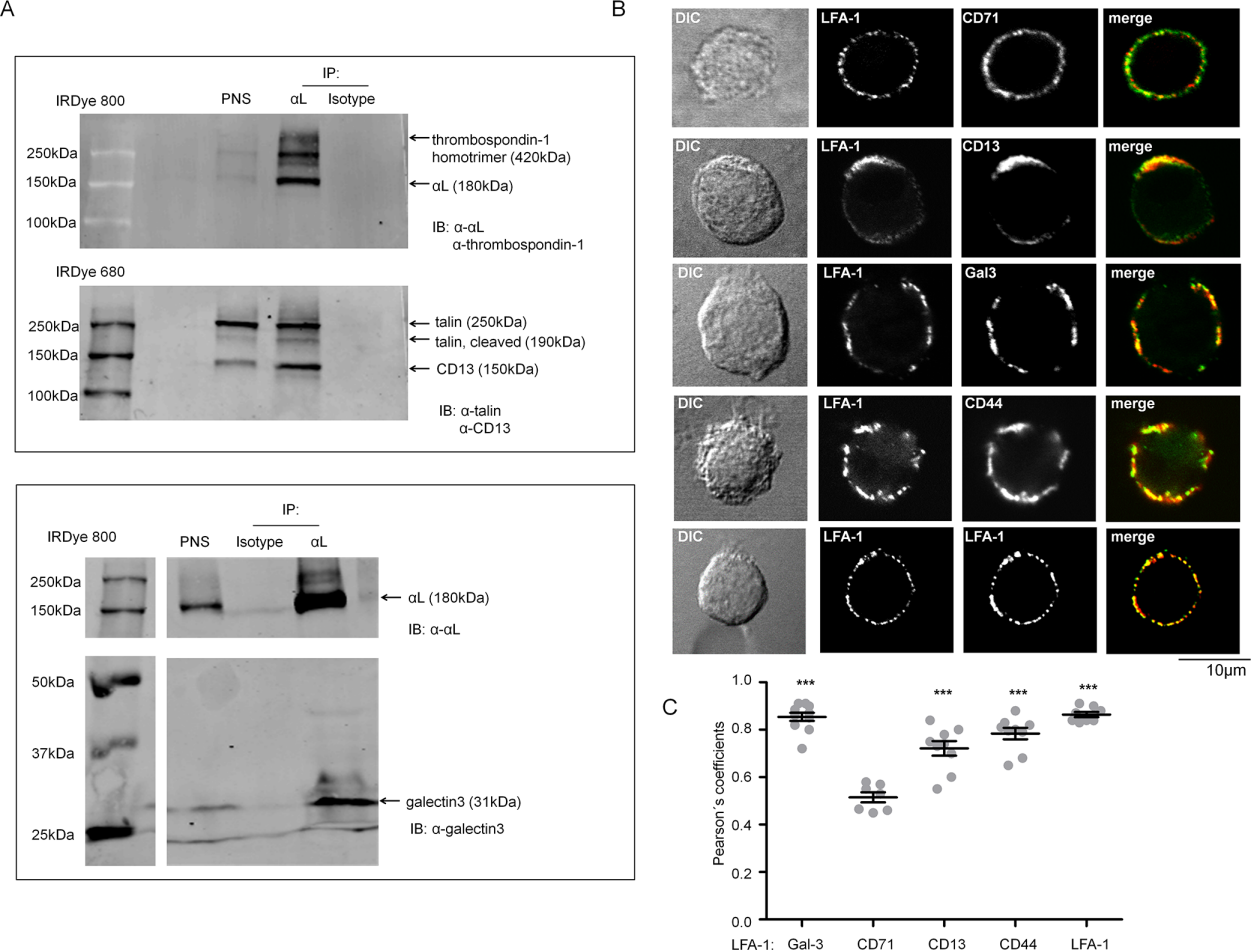
Validation of MS results by WB and proximity study by confocal microscopy for selected proteins. (A) Protein complexes of LFA-1, thrombospondin-1, talin-1, CD13 (top) and galectin-3 (bottom) in imDCs (day6) were co-immunoprecipitated with LFA-1. LFA-1 was enriched using mAb (clone SPV-L7) directed against αL (CD11a). mIgG1 coated beads were included as control IP. PNS: post nuclear supernatant. Samples were analysed in non-reducing conditions. (B) Confocal microscopy analysis of co-capping of LFA-1 and galectin-3, CD44 and CD71 on imDCs (day6). Receptor co-capping and staining were performed as described in *Material and Methods*. Antibodies against LFA-1 (clones: NKI-L15 and TS2/4) and CD71 are positive and negative markers for co-localization, respectively. Results are representatives of multiple cells per condition (n>10.) in two independent experiments. (C) To quantify the degree of co-localization between LFA-1 and binding candidates, Pearson´s coefficient was calculated. The values can vary between 0 and 1 (1 = 100% colocalization). P-values were compared to co-capping of LFA-1 with CD71 by two-tailed t-test, *** <0.001. Co-capping and staining were performed as described in Materials and Methods.

Next, we sought to validate some of the identified candidate proteins by Western Blotting (WB). As the formation of LFA-1 nanoclusters and LFA-1 activity is lost during DC differentiation [13,15], we were specifically interested in proteins that interact with LFA-1 in the PM rather than those that may interact with LFA-1 in intracellular organelles. Of the proteins that passed our enrichment criteria we selected the potential LFA-1 interaction partners galectin-3 (LGALS3), thrombospondin-1 (THBS1), CD13 (ANPEP) and CD44, as well as the established binding partners kindlin-3 (FERMT3) and talin-1 (TLN1) for further study. WB analysis was in agreement with the results from the MS, demonstrating co-IP of thrombospondin-1, talin-1, CD13 and galectin-3 with LFA-1 in DCs (Fig 3A).

### Proximity of LFA-1 to CD13, CD44 and galectin-3 at the PM

Not all antibodies available to us allowed confirmation of interaction with LFA-1 by Co-IP and WB. As an alternative approach to validate the relation of these proteins to LFA-1, we also examined the proximity of LFA-1 with the PM receptors and PM- associated proteins CD13, CD44 and galectin-3 by mAb-induced capping and confocal microscopy (Fig 3B). When co-capping was induced, LFA-1 strongly colocalized with galectin-3 in the PM with a Pearson´s coefficient of 0.85 (Fig 3B and 3C). CD13 and CD44 localization after capping also correlated significantly with LFA-1 (Pearson´s coefficients 0.8 and 0.72 respectively) in contrast to the non-LFA-1 associated protein CD71 (0.52). Thus, our data confirm that CD13, galectin-3 and CD44 reside in proximity to LFA-1 in the PM, however further experiments are required to elucidate their role in LFA-1 function and whether they interact directly or indirectly with LFA-1.

### In-silico reconstruction of the LFA-1 protein-protein interaction network in DCs

**Fig 4:**
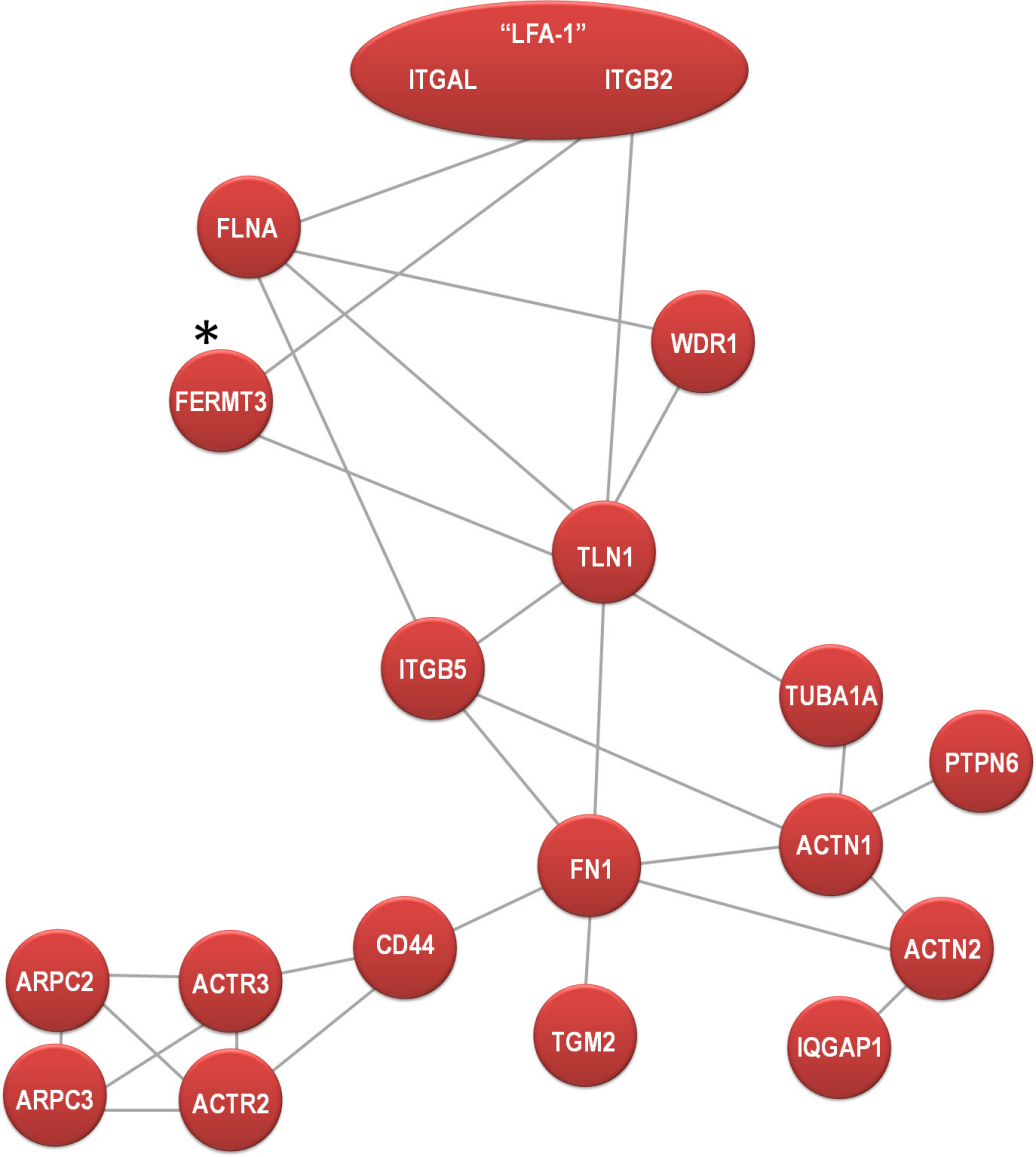
Protein-protein interaction of directly interacting LFA-1 binding candidates derived from DCs in stringent lysis conditions. A network was generated by uploading the protein names to the database of functional protein interactions (STRING v9.05) and retrieving experimentally proven direct protein-protein interactions. The resulting network was drawn by the authors. Based on our MS data, we could retrieve a high confidence network (score 0.6) of 19 directly interacting nodes (Fig 5), enriched in 31 connections. * indicates an interaction that was not present in the STRING database (version 9.1) with experimental support, but this node and interactions were added by the authors based on the current literature [27].

Having confirmed the validity of our proteomics data by WB and microscopy, we next aimed to reconstruct the steady-state LFA-1 interactome in primary human DCs from the list of 83 proteins (including α and β chain of LFA-1) identified as strong LFA-1 interactors (Table 1). To construct a PPI network we next interrogated the database of functional protein interactions (STRING v9.05) for experimentally proven protein-protein interactions. For better visualization of proteins involved in integrin function we excluded 19 proteins corresponding to ribosomal and histone complexes, which likely represent a pollution of these highly abundant proteins. The remaining 64 proteins were uploaded to STRING and a network of 19 directly interacting proteins could be formed (Fig 4; confidence score 0.6), potentially representing the core network of strong LFA-1 interactors in DCs. This network contained 31 connections, resulting in an average of 0.48 connections per protein in our list of 64 LFA-1 binding partners (Table 1).

**Fig 5:**
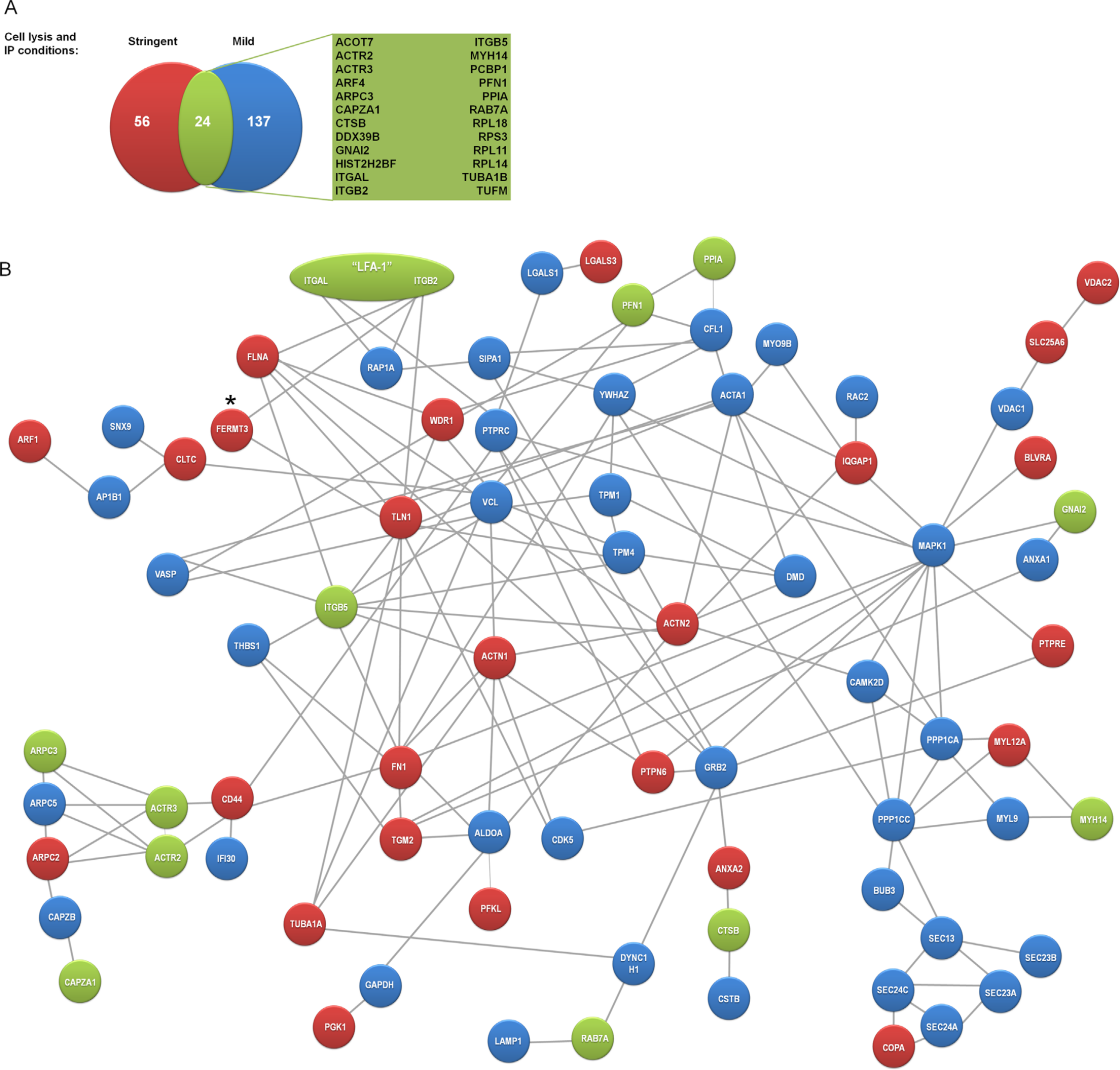
Protein-protein interaction network of directly interacting LFA-1 binding candidates derived from DCs. (A) Comparison of immunoprecipitated LFA-1 from day 6 imDCs in mild and stringent lysis conditions. Venn diagrams of proteins identified in DCs in stringent (red) and mild (blue) IP conditions. Numbers of identified proteins, as well as common proteins (yellow) are indicated. (B) A network of LFA-1 (heterodimer formed by an αL (ITGAL) and β2 (ITGB2) chain) binding partners was generated by fusing the data sets derived from mild and stringent IP conditions in DCs, uploading the protein names to the database of functional protein interactions (STRING v9.05) and retrieving experimental proven direct protein-protein interactions. (ribosomal and histone complexes were removed for better visualization of proteins involved in integrin function). The resulting network was redrawn by the authors. Based on our MS data, we could construct a high confidence network (score 0.6) containing 78 nodes and 154 connections. Blue nodes represent proteins identified in mild lysis conditions, and red nodes represent proteins identified in stringent lysis conditions. Green nodes represent proteins identified both in mild and stringent IP condition. * indicates an interaction that was not present in the STRING database (version 9.1) with experimental support, but this node and interactions were added by the authors based on the current literature [27].

As a source of additional nodes to further construct the LFA-1 network we complemented our high confident IP performed under stringent lysis conditions with IPs of DC LFA-1 performed in the presence of a mild detergent. The IP performed in mild conditions resulted in the same IP efficacy as stringent IP condition, as confirmed by WB (data not shown) and peptide count (S2 Table). Our aim was to use the less stringently LFA-1 associated proteins as a “LFA-1-interactome-enriched” fishing pool to extract indirect higher order binding partners for the proteins retrieved under stringent conditions. For maximal sensitivity in detecting potential LFA1 interactors, we applied slightly less stringent criteria for unique proteins now demanding 2 unique peptides in the specific IP or at least 2.5 fold iBAQ enrichment over control IP in one of the performed experiments. Applying these enrichment criteria for the mild IP condition, we now identified 171(IPI) proteins (mapping to 161 HGNC symbols) as LFA-1 binding candidates (S1 Table), of which 24 proteins were also identified in stringent IP conditions (Fig 5A).

To construct a PPI network we next fused the data sets derived from mild and stringent IP conditions (again after removal of ribosmal and histone complex proteins) and interrogated the database of functional protein interactions (STRING v9.05). We then constructed a highly confident PPI network (confidence score 0.6) (Fig 5B) composed of 78 proteins and 154 interactions. The network links cytoskeletal, signaling and scaffolding proteins to both secreted and PM associated proteins, including the known interaction partners Rap1a (RAP1A), kindlin-3, talin-1 and vinculin (VCL), as well as the novel LFA-1 interactors galectin-3 and thrombospondin-1 confirmed in this study (Fig 5B). Of all proteins in the network, vinculin represents the best connected node with 13 connections, followed by talin-1 with 12 connections and actin (Acta1) and growth factor receptor-bound protein 2 (GRB2) with 10 connections, suggesting a fundamental role of these proteins in LFA-1 function in DCs (Fig 5B). Interestingly, based on STRING, only 6 proteins directly interacted with the α or β chain of LFA-1, while the majority of protein interactions are of secondary-tertiary, or higher degree nature, suggesting that large protein complexes are associated with LFA-1 in the steady-state in DCs

Addition of the proteins derived uniquely by mild IP conditions to the initial LFA-1 network resulted in a larger network with 78 proteins directly connected by 154 interactions (Fig 5B) and this exercise thus doubled the average connection of the identified LFA-1 binding partners from 0.48 (for stringent IP condition only) to 0.96 when the combined list was uploaded (159 proteins, after removal of ribosomal and histone complexes). The obtained network contained 3.5 fold more connections than could be expected based on an equally sized set of random proteins predicted by STRING. Taken together, this indicates that we have constructed a network of proteins that have a high potential to act together to regulate LFA-1 function in DCs (Fig 5B).

### LFA-1 interactome in monocytes

**Fig 6:**
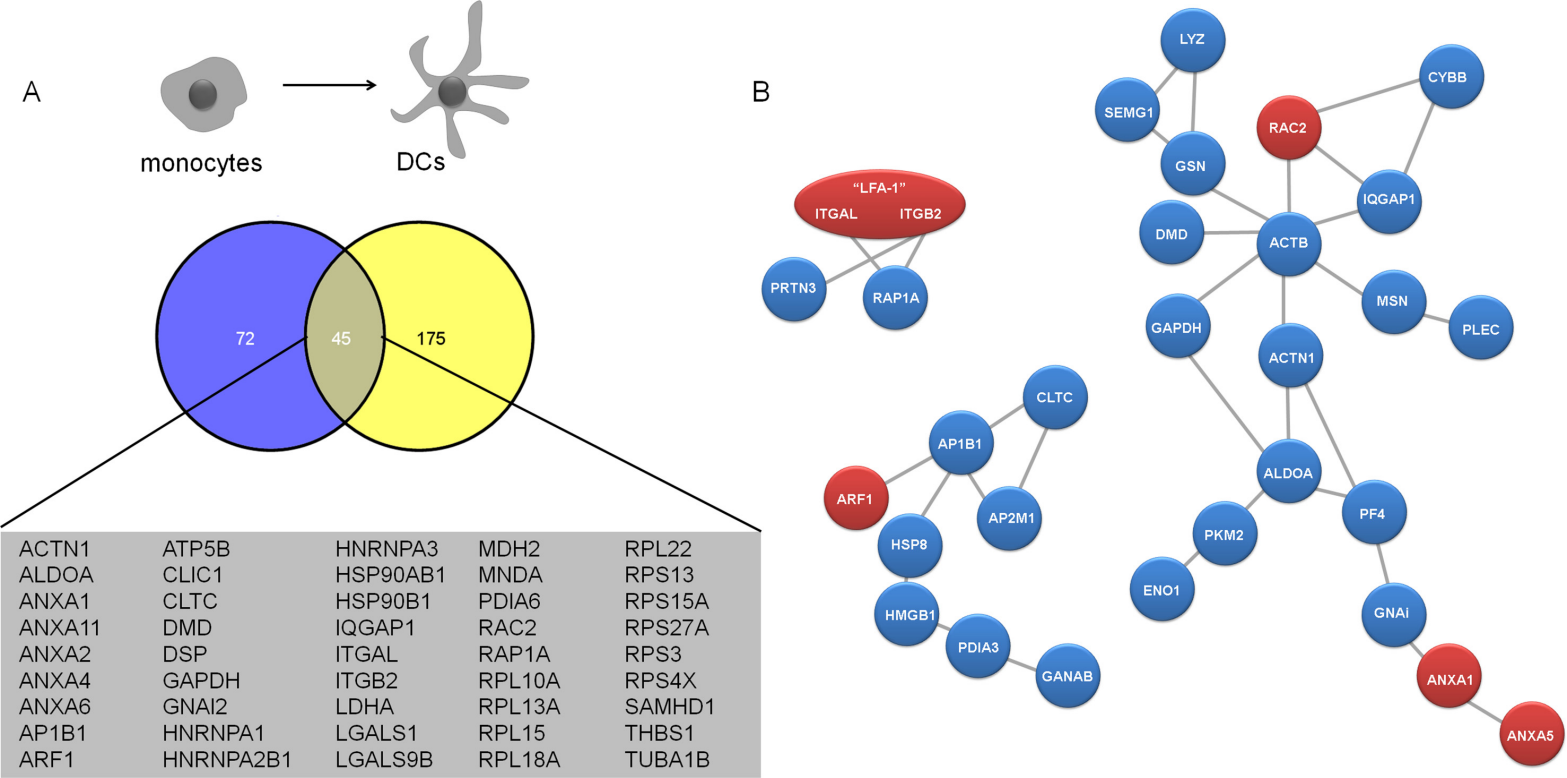
Comparison of total LFA-1 binding partners (derived from mild and stringent lysis conditions) in monocytes and DCs. (A) Venn diagrams of proteins identified in monocytes (blue) and DCs (yellow). Numbers of identified proteins, as well as common proteins are indicated. (B) PPI network of directly interacting LFA-1 (heterodimer formed by and αL (ITGAL) and β2 (ITGB2) chain) binding partners derived from monocytes in stringent lysis and mild lysis conditions. A network was generated by uploading the protein names to the database of functional protein interactions (STRING v9.05) and retrieving experimental proven direct protein-protein interactions. The resulting network was drawn by the authors. Based on our MS data, we could retrieve 3 high confidence networks (score 0.6), with a maximum of 19 directly interconnected nodes. Blue nodes represent proteins identified in mild lysis conditions, and red nodes represent proteins identified in stringent lysis conditions.

Having reconstructed a LFA-1 PPI network in primary DCs from our MS data, we were next wondering how specific this network was for DCs and whether the changes with respect to LFA-1 function, activation state and cell surface organization in DCs were reflected in the repertoire of LFA-1 binding partners. To this end, we used a comparable IP strategy on primary human monocytes, which we use as precursors to generate DCs. The efficacy of the LFA-1 IP from monocytes was similar to the LFA-1 IPs performed in DCs (S2 Table). As potential interaction partners for LFA-1 in monocytes we identified in total (i.e. both mild and stringent conditions) 117 unique proteins mapped to HGNC symbol (S2 Table), slightly less than in DCs, of which 45 were shared between monocytes and DCs, including the known integrin interaction partner Rap1A and the scaffolding protein IQGAP1 (Fig 6A). Next, we interrogated STRING to build a PPI network based on the LFA-1 binding candidates identified in monocytes. This high confidence LFA- PPI network (score of 0.6) only contained 19 proteins forming a coherent network by 24 connections, resulting in an average connection of 0.23 per protein detected in mild and stringent IP conditions in monocytes (104 proteins after removal of ribosomal and histone complexes) (Fig 6B). Overall, we identified more LFA-1 interactors in DCs, suggesting that the underlying LFA-1 PPI network may be more complex in DCs.

**Fig 7:**
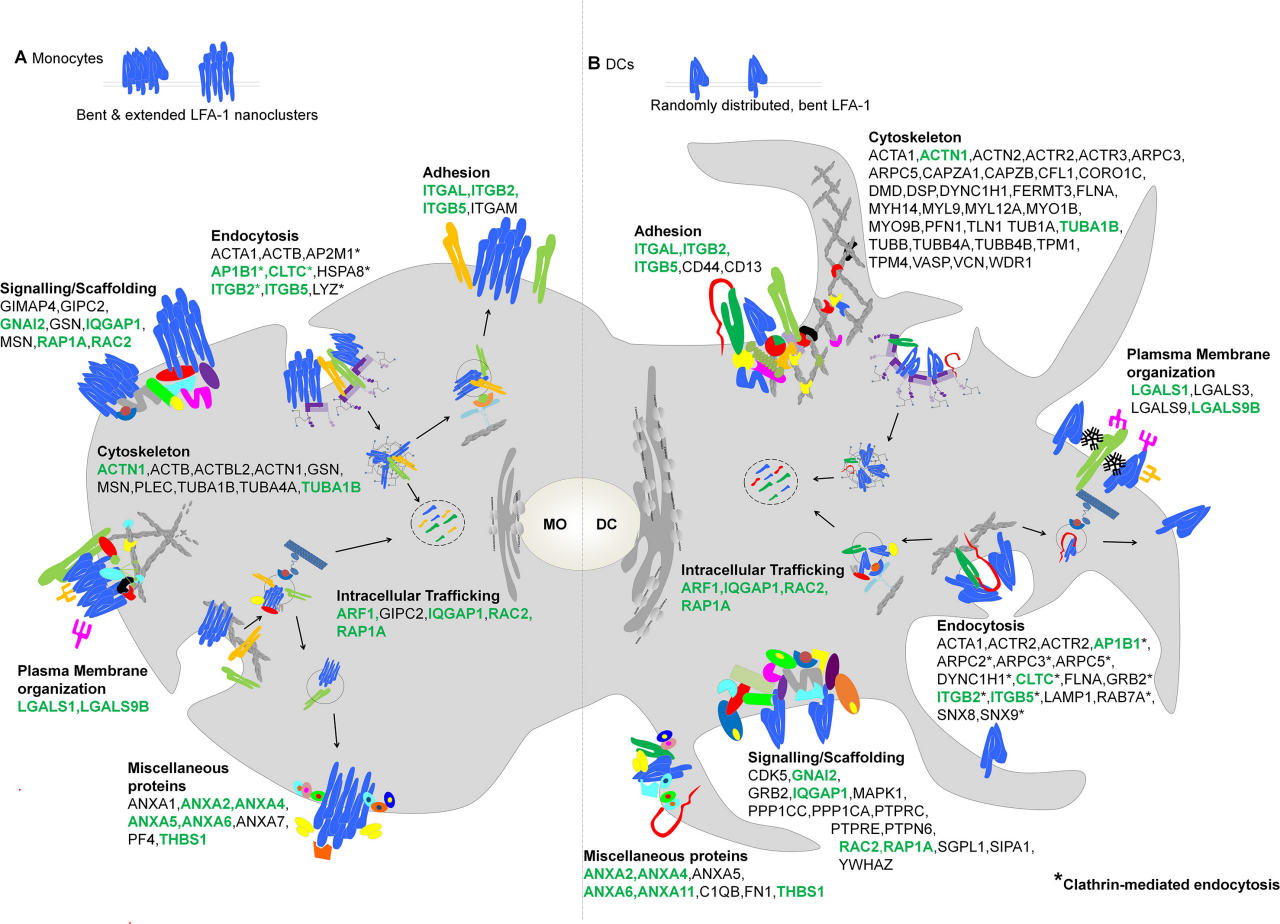
Model for the Spatio-Functional Regulation of LFA-1 in monocytes and DCs. Schematic representation of LFA-1 binding partners (described by gene symbols) in monocytes and DCs, selected from the full lists of enriched proteins identified in these cell types in this study (Table 1, S1 and S2 Tables). The authors selected the binding candidates according to their established or potentially novel role in integrin function and cell adhesion, based on the current literature. Candidates were grouped into categories according to their potential role and/ or cellular localization. In monocytes, in the absence of ligand, LFA-1 (heterodimer formed by an αL (ITGAL) and β2 (ITGB2) chain) is functionally active and forms nanoclusters consisting of extended or inactive (bent) LFA-1 molecules in the PM (A). During differentiation of monocytes towards DCs (B) LFA-1 activity is lost (all molecules are in the bent conformation) and LFA-1 shows a random PM distribution. LFA-1 binding partners identified in both monocytes and DCs are highlighted in green.

Together, the here described established as well as potentially novel interaction partners of LFA-1 may map the way to a better understanding of the regulatory mechanisms acting on LFA-1 in DCs and monocytes as depicted in the cartoon (Fig 7). The interaction partners identified may function to steer LFA-1 intracellular trafficking, plasma membrane organization, activation and signalling and provide a valuable resource for future LFA-1 research.

## Discussion

We have previously shown that LFA-1 undergoes dramatic changes in function and cell surface organization during *in-vitro* differentiation of moDCs, changing from a discrete nanoclusters organisation and extended conformation to randomly distributed inactive LFA-1 [13,15]. LFA-1 adhesive behavior and motility on DCs however can be rapidly changed by the chemokine CCL21 [15,28]. The differential regulation of LFA-1 on these cell types may reflect the different circumstances in which LFA-1 has to act in each case. To aid monocyte extravasation into tissues, pro-adhesive LFA-1 nanoclusters form high-affinity bonds to endothelial-presented ICAM-1 which are stabilized by the high shear stress in the blood stream [29]. On DCs, LFA-1 likely plays a role during trans-lymphatic migration to the lymph nodes, where low shear stress values prevail and a chemotactic gradient of CCL21 is present [30]. The demands of these different environments require distinct regulatory mechanisms to allow optimal LFA-1 function in each case. Here we present the first effort to identify proteins specifically associated with LFA-1 in each cell type, to get more insight into the molecules that may regulate LFA-1 function.

Together a wealth of small scale biochemical studies have so far identified more than 40 proteins that directly interact with either the α or β chain of LFA-1 from different cell types [27,31,32]. Large scale efforts to characterize the proteomes of integrin containing adhesion complexes, including focal adhesions, invadopodia and podosomes however showed that ligand-induced adhesion structure complexes may consist of up to 200 different proteins constituting several layers of interaction [5–9], and we estimate that this number only represents the tip of the iceberg [11]. In the present study we set out to characterize the broad LFA-1 adhesome prior to ligand binding by selective IP of the αL chain of LFA-1 coupled to highly sensitive MS analysis. We identified a set of proteins associated to LFA-1 in human monocyte-derived DCs suggesting a pre-organization of LFA-1 in molecular platforms even in the absence of ligand binding. The choice of lysis buffer greatly influenced the number of proteins identified in DCs in our study. As expected, we identified more binding partners in mild lysis conditions. According to our protein-protein interaction analysis, of the identified binding partners only 6 proteins directly interacted with the α or β chain of LFA-1 and only 20 proteins formed a coherent higher degree (e.g. secondary, tertiary) network in stringent lysis conditions, potentially representing a core network of strong LFA-1 binding partners in DCs. Potential binding partners derived by mild IP condition greatly enhanced the network of LFA-1 binding partners in number and interconnectivity, suggesting that the pre-organized molecular platforms associated with LFA-1 in DCs may largely be sustained by proteins only indirectly associated to LFA-1. Nonetheless, by their close proximity to LFA-1, these proteins may be very important for LFA-1 function in DCs.

Our MS data reveals that both on DCs and on monocytic precursors LFA-1 is associated with a set of signaling and cytoskeletal proteins known to act downstream of adhesion receptors in leukocytes, such as Rap1A [33], Rac2 [34], GNAi2 [35], IQGAP1 [36], actin [37], tubulin [38] and thus suggesting a fundamental role of these proteins for LFA-1 function regardless of the cell type. In addition we have identified a distinct set of LFA-1 interacting proteins only in DCs. These proteins may facilitate the instantaneous activation and immobilization of LFA-1 in response to CCL21 [15,28] as well as an altered organization of LFA-1 on the PM [13]

The most proximal signaling event leading to integrin activation is the binding of the cytosolic actin-interacting proteins kindlin-3 and talin-1, causing the separation of the integrin cytosplasmic tails and TM region, leading to integrin extension [39,40]; therefore these proteins are classically thought to interact only with active (extended) integrins. Strikingly, we identified the interaction of both of these proteins with LFA-1 in DCs, where LFA-1 is mostly inactive (which was confirmed by flow cytometry). In line with our finding, it was recently reported that talin-1 colocalized with LFA-1 in DCs prior to ligand binding and that CCL21 did not increase this interaction. Ligand binding that further stabilized chemokine-induced LFA-1 activation however did induce an increase of talin binding [28]. Others have also reported interaction of full-length talin with inactive integrins in neutrophils [41]. Our data is in line with the finding that talin-1 does not exclusively interact with active integrins and suggests a more complex role of talin-1 and potentially kindlin-3 in LFA-1 regulation. Unexpectedly we could not detect talin-1 and kindlin-1 in our MS data for monocytes that have a large amount of active LFA-1 on their surface. Possibly, in monocytes ligand binding is a prerequisite for the binding of talin-1 and kindlin-3 to LFA-1.

Interestingly, we specifically found the following proteins associated with integrin signalling in the LFA-1 pull-down fraction in DCs: Protein tyrosine phosphatase, receptor type C (PTPRE, CD148), type E (PTPRC, CD45) and 14-3-3 (YWHAZ). CD148 and CD45 regulate membrane-associated non-receptor Src family kinases (SFK), which can directly interact with the integrin cytoplasmic tail and regulate integrin affinity and outside-in signaling [42,43]. 14-3-3 proteins are dimers, and the 14-3-3 αβ and δζ isoforms were previously shown to associate with the cytoplasmic tail of the β chain of LFA-1 and to regulate LFA-1–mediated cell adhesion and spreading [44]. We also identified Spa-1 (SIPL-1), a Rap GTPase-activating protein reported as negative regulator of cell adhesion, which could thus play a role in the inactivation of LFA-1 during differentiation into DCs [45].

Furthermore we found a more abundant presence of proteins in the DC IP that may link LFA-1 to the cytoskeleton and regulate actin dynamics, including components of the Arp2/3 complex, F-actin capping proteins, VASP, cofilin, coronin, WDR1 and vinculin. Vinculin interacts directly with actin and talin through binding sites in the talin rod. Moreover, vinculin, WDR1, cofilin and coronin appear to regulate actin turnover via Arp2/3 [46,47]. The specific association of these cytoskeletal proteins with LFA-1 in DCs suggests a higher connectivity of LFA-1 to the cytoskeleton, possibly facilitating efficient DC migration [48]. In this respect, we have previously shown that LFA-1 mobility within the PM of monocytes is an important requisite for reinforcing binding to ICAM-1 [49]. An increased association of LFA-1 with the cytoskeleton in DCs could prevent mobile LFA-1 from outgrowing ligand-induced adhesion sites, thereby hampering LFA-1-mediated adhesion, as observed during DC differentiation [13,15].

CD13/Aminopeptidase N (ANPEP) is highly expressed in both monocytes and DCs (data not shown), yet only precipitated together with LFA-1 in DCs. We could confirm its close proximity to LFA-1 on the DC surface and thus this protein likely represents a novel LFA-1 interaction partner. The association of integrins with metalloproteases has been shown to play a role in degrading ECM and to modulate receptor activity [50]. CD13 is a metallo-exopeptidase with demonstrated involvement in leukocyte activation and migration, by triggering calcium influx, cytoskeletal rearrangements and integrin activation [51]. Its selective association with LFA-1 in DC thus makes it an interesting candidate for further investigation.

We also identified several proteins of the galectin family including galectin-1–3 and -9 as potential LFA-1 interaction partners. Galectins are carbohydrate binding proteins associated to many cellular processes including signaling, migration, endocytosis and cell surface organization via their scaffolding properties [52]. Of these galectins, galectin-3 was only detected in the DCs IP and therefore of specific interest. We validated its association with LFA-1 using both WB and microscopy demonstrating for the first time its connection with LFA-1. Galectin-3 uniquely forms pentamers allowing it to form glycan lattices and has been shown previously to bind and regulate the α_3_β_1_ integrin, facilitating lamellipodia formation by activating Rac1 signaling in epithelial cells [53]. Intriguingly, galectin-3 was recently also demonstrated to regulate a novel clathrin-independent, glycosphingolipid dependent endocytic route taken by N-glycosylated cargo including the β1 integrins and CD44 [54]. Based on these data, it is tempting to speculate that the specific interaction of galectin-3 with LFA-1 in DCs could point towards a selective usage of this endocytic route in DCs. The co-precipitation of CD44 with LFA-1 and the proximal distribution of these molecules on the DC PM could reflect their shared usage of this route. In agreement with such a differential regulation of endocytosis in DCs and monocytes we found LFA-1 in monocytes to co-precipitate with the clathrin-adaptor subunit AP2M1 that is part of the AP-2 complex regulating clathrin mediated endocytosis from the PM [55]. Alternatively (or concomitantly) the scaffolding properties of galectin-3 (and possibly other galectins) could play a role in the altered membrane organization of LFA-1 on DCs with respect to monocytes.

Overall, the DC-LFA-1PPI network constructed from our identified interaction partners shows a high level of connectivity, increasing the likelihood that some of these interactions also occur *in vivo*. However, we should be aware that our reconstruction of the DC-LFA-1 interactome likely is not complete and therefore the network is only an approximation of the reality. The complexity of our samples will have favored the detection of largest, most abundant, most hydrophilic proteins resistant to cell lysis. Although our samples contained many established interaction partners of LFA-1 and we could already validate the proximity of LFA-1 to the newly identified interaction partners CD13, galectin-3 and CD44, further biochemical and functional analysis will be needed to validate the remaining potential LFA-1 binding partners. Nonetheless, the here presented set of possible LFA-1 interaction partners provides a valuable resource in the search for mechanistic insight into the regulation of LFA-1 function in DCs and monocytes.

Taken together, our results strongly support the notion that in DCs at steady-state LFA-1 resides in the vicinity of a great number of signalling, cytoskeletal and PM proteins that are involved in inside-out and outside-in signalling, PM organisation as well as intracellular trafficking. Such a preorganization can greatly facilitates rapid modulation of LFA-1 function in DCs, as observed upon exposure to CCL21, to quickly adapt to the requirements of the dynamically changing DC environment. Further (MS) experiments that lie beyond the scope of the current manuscript are needed to answer the intriguing question whether CCL21 stimulation modifies the LFA-1 interactome or mostly acts by changing the activation state of proteins already present in pre-existing molecular platforms associated with LFA-1 in DCs.

## Supporting Information

S1 Table Protein binding partners identified in mild IP conditions in DCs. The criteria for protein selection were the following: proteins were at least 2.5 fold enriched over the isotype-type matched control IP (based on the normalized IBAQ value), or were specifically detected in the LFA-1 IP with a minimum of 2 unique peptides. HGNC symbols and protein names were assigned to the International Protein Index (IPI) in IPA. Italic indicates proteins detected in multiple experiments and IBAQ values and peptide counts were taken from the best experiment.
(DOCX)

S2 Table Protein binding partners identified in mild (top) and stringent (bottom) IP conditions in monocytes. The criteria for protein selection were the following: proteins were at least 2.5 fold enriched over the isotype-type matched control IP (based on the normalized IBAQ value), or were specifically detected in the LFA-1 IP with a minimum of 2 and 3 unique peptides in mild and stringent IP conditions, respectively. Proteins were mapped to HGNC and International Protein Index (IPI) identifiers. Italic indicates proteins detected in multiple experiments and IBAQ values and peptide counts were taken from the best experiment.
(DOCX)
