# Supplementary material for: Proteome Based Construction of the Lymphocyte Function-Associated Antigen 1 (LFA-1) Interactome in Human Dendritic Cells
Source: PLoS One. 2016 Feb 18;11(2):e0149637. doi: 10.1371/journal.pone.0149637 (PMC4758637; doi:10.1371/journal.pone.0149637)
Supplement: S1 Table — The criteria for protein selection were the following: proteins were at least 2.5 fold enriched over the isotype-type matched control IP (based on the normalized IBAQ value), or were specifically detected in the LFA-1 IP with a minimum of 2 unique peptides. HGNC symbols and protein names were assigned to the International Protein Index (IPI) in IPA. Italic indicates proteins detected in multiple experiments and IBAQ values and peptide counts were taken from the best experiment. (DOCX) [file pone.0149637.s001.docx]

**S1 Table:**

|  | **Mild IP conditions**  **Protein AcNR** | **HGNC symbol** | **Protein Name (IPA)** | **normalized IBAQ control IP** | **normalized IBAQ LFA-1 IP** | **max peptides control** | **max peptides LFA-1 IP** | **fold change (LFA-1 IP/Control IP)** |
| --- | --- | --- | --- | --- | --- | --- | --- | --- |
| 1 | *IPI00291792* | *ITGB2* | *integrin, beta 2* | *nd* | *104.53* | *0* | *25* | *LFA-1 IP specific* |
| 2 | IPI00328257 | AP1B1 | adaptor-related protein complex 1, beta 1 subunit | nd | 2.10 | 0 | 13 | LFA-1 IP specific |
| 3 | IPI00604620 | NCL | nucleolin | nd | 4.73 | 0 | 7 | LFA-1 IP specific |
| 4 | *IPI00796333* | *ALDOA* | *aldolase A, fructose-bisphosphate* | *nd* | *1.21* | *0* | *7* | *LFA-1 IP specific* |
| 5 | *IPI00009904* | *PDIA4* | *protein disulfide isomerase family A, member 4* | *nd* | *0.95* | *0* | *6* | *LFA-1 IP specific* |
| 6 | IPI00215965 | HNRNPA1 | heterogeneous nuclear ribonucleoprotein A1 | nd | 1.72 | 0 | 5 | LFA-1 IP specific |
| 7 | IPI00216587 | RPS8 | ribosomal protein S8 | nd | 11.21 | 0 | 5 | LFA-1 IP specific |
| 8 | IPI00413324 | RPL17 | ribosomal protein L17 | nd | 4.64 | 0 | 5 | LFA-1 IP specific |
| 9 | IPI00018931 | VPS35 | vacuolar protein sorting 35 homolog (S. cerevisiae) | nd | 0.50 | 0 | 4 | LFA-1 IP specific |
| 10 | IPI00025091 | RPS11 | ribosomal protein S11 | nd | 17.00 | 0 | 4 | LFA-1 IP specific |
| 11 | IPI00027270 | RPL26 | ribosomal protein L26 | nd | 14.76 | 0 | 4 | LFA-1 IP specific |
| 12 | IPI00182533 | RPL28 | ribosomal protein L28 | nd | 5.41 | 0 | 4 | LFA-1 IP specific |
| 13 | IPI00216691 | PFN1 | profilin 1 | nd | 6.21 | 0 | 4 | LFA-1 IP specific |
| 14 | IPI00301936 | ELAVL1 | ELAV like RNA binding protein 1 | nd | 2.92 | 0 | 4 | LFA-1 IP specific |
| 15 | IPI00872940 | RPL30 | ribosomal protein L30 | nd | 10.87 | 0 | 4 | LFA-1 IP specific |
| 16 | IPI00940950 | RPL10 | ribosomal protein L10 | nd | 8.28 | 0 | 4 | LFA-1 IP specific |
| 17 | IPI00005171 | HLA-DRA | major histocompatibility complex, class II, DR alpha | nd | 7.09 | 0 | 3 | LFA-1 IP specific |
| 18 | IPI00021327 | GRB2 | growth factor receptor-bound protein 2 | nd | 1.34 | 0 | 3 | LFA-1 IP specific |
| 19 | IPI00307162 | VCL | vinculin | nd | 0.12 | 0 | 3 | LFA-1 IP specific |
| 20 | IPI00395627 | CACYBP | calcyclin binding protein | nd | 1.50 | 0 | 3 | LFA-1 IP specific |
| 21 | IPI00794659 | RPS20 | ribosomal protein S20 | nd | 26.36 | 0 | 3 | LFA-1 IP specific |
| 22 | IPI00926935 | GNAI2 | guanine nucleotide binding protein (G protein), alpha inhibiting activity polypeptide 2 | nd | 1.46 | 0 | 3 | LFA-1 IP specific |
| 23 | IPI00010270 | RAC2 | ras-related C3 botulinum toxin substrate 2 (rho family, small GTP binding protein Rac2) | nd | 1.89 | 0 | 3 | LFA-1 IP specific |
| 24 | IPI00004524 | GCA | grancalcin, EF-hand calcium binding protein | nd | 0.23 | 0 | 2 | LFA-1 IP specific |
| 25 | IPI00007752 | TUBB4B | tubulin, beta 4B class IVb | nd | 1.89 | 0 | 2 | LFA-1 IP specific |
| 26 | IPI00010153 | RPL23 | ribosomal protein L23 | nd | 2.90 | 0 | 2 | LFA-1 IP specific |
| 27 | IPI00013468 | BUB3 | BUB3 mitotic checkpoint protein | nd | 0.08 | 0 | 2 | LFA-1 IP specific |
| 28 | IPI00013917 | RPS12 | ribosomal protein S12 | nd | 2.24 | 0 | 2 | LFA-1 IP specific |
| 29 | IPI00016342 | RAB7A | RAB7A, member RAS oncogene family | nd | 0.62 | 0 | 2 | LFA-1 IP specific |
| 30 | IPI00018206 | GOT2 | glutamic-oxaloacetic transaminase 2, mitochondrial | nd | 7.09 | 0 | 2 | LFA-1 IP specific |
| 31 | IPI00019345 | RAP1A | RAP1A, member of RAS oncogene family | nd | 0.95 | 0 | 2 | LFA-1 IP specific |
| 32 | IPI00021167 | PRKRA | protein kinase, interferon-inducible double stranded RNA dependent activator | nd | 0.24 | 0 | 2 | LFA-1 IP specific |
| 33 | IPI00021266 | RPL23A | ribosomal protein L23a | nd | 4.08 | 0 | 2 | LFA-1 IP specific |
| 34 | IPI00024911 | ERP29 | endoplasmic reticulum protein 29 | nd | 1.01 | 0 | 2 | LFA-1 IP specific |
| 35 | IPI00025277 | PDCD6 | programmed cell death 6 | nd | 1.06 | 0 | 2 | LFA-1 IP specific |
| 36 | IPI00028055 | TMED10 | transmembrane emp24-like trafficking protein 10 (yeast) | nd | 3.28 | 0 | 2 | LFA-1 IP specific |
| 37 | IPI00031691 | RPL9 | ribosomal protein L9 | nd | 3.56 | 0 | 2 | LFA-1 IP specific |
| 38 | IPI00032139 | SERPINB9 | serpin peptidase inhibitor, clade B (ovalbumin), member 9 | nd | 0.16 | 0 | 2 | LFA-1 IP specific |
| 39 | IPI00032561 | CAB39 | calcium binding protein 39 | nd | 0.07 | 0 | 2 | LFA-1 IP specific |
| 40 | IPI00165230 | DAZAP1 | DAZ associated protein 1 | nd | 0.82 | 0 | 2 | LFA-1 IP specific |
| 41 | IPI00215719 | RPL18 | ribosomal protein L18 | nd | 2.80 | 0 | 2 | LFA-1 IP specific |
| 42 | IPI00218606 | RPS23 | ribosomal protein S23 | nd | 5.34 | 0 | 2 | LFA-1 IP specific |
| 43 | IPI00218782 | CAPZB | capping protein (actin filament) muscle Z-line, beta | nd | 0.78 | 0 | 2 | LFA-1 IP specific |
| 44 | IPI00218918 | ANXA1 | annexin A1 | nd | 0.20 | 0 | 2 | LFA-1 IP specific |
| 45 | IPI00219757 | GSTP1 | glutathione S-transferase pi 1 | nd | 1.33 | 0 | 2 | LFA-1 IP specific |
| 46 | IPI00220278 | MYL9 | myosin, light chain 9, regulatory | nd | 1.61 | 0 | 2 | LFA-1 IP specific |
| 47 | IPI00221327 | RTCA | RNA 3'-terminal phosphate cyclase | nd | 2.29 | 0 | 2 | LFA-1 IP specific |
| 48 | IPI00295741 | CTSB | cathepsin B | nd | 0.85 | 0 | 2 | LFA-1 IP specific |
| 49 | IPI00296099 | THBS1 | thrombospondin 1 | nd | 0.08 | 0 | 2 | LFA-1 IP specific |
| 50 | IPI00301058 | VASP | vasodilator-stimulated phosphoprotein | nd | 0.78 | 0 | 2 | LFA-1 IP specific |
| 51 | IPI00304181 | AGO3 | argonaute RISC catalytic component 3 | nd | 0.13 | 0 | 2 | LFA-1 IP specific |
| 52 | IPI00401264 | ERP44 | endoplasmic reticulum protein 44 | nd | 0.38 | 0 | 2 | LFA-1 IP specific |
| 53 | IPI00411706 | ESD | esterase D | nd | 0.38 | 0 | 2 | LFA-1 IP specific |
| 54 | IPI00412607 | RPL35 | ribosomal protein L35 | nd | 3.77 | 0 | 2 | LFA-1 IP specific |
| 55 | IPI00413451 | SERPINB6 | serpin peptidase inhibitor, clade B (ovalbumin), member 6 | nd | 0.23 | 0 | 2 | LFA-1 IP specific |
| 56 | IPI00477992 | C1QB | complement component 1, q subcomponent, B chain | nd | 1.27 | 0 | 2 | LFA-1 IP specific |
| 57 | IPI00550234 | ARPC5 | actin related protein 2/3 complex, subunit 5, 16kDa | nd | 1.08 | 0 | 2 | LFA-1 IP specific |
| 58 | IPI00641829 | DDX39B | DEAD (Asp-Glu-Ala-Asp) box polypeptide 39B | nd | 0.09 | 0 | 2 | LFA-1 IP specific |
| 59 | IPI00827625 | CAMK2D | calcium/calmodulin-dependent protein kinase II delta | nd | 0.71 | 0 | 2 | LFA-1 IP specific |
| 60 | IPI00853598 | SEC13 | SEC13 homolog (S. cerevisiae) | nd | 2.91 | 0 | 2 | LFA-1 IP specific |
| 61 | IPI00915363 | RPS24 | ribosomal protein S24 | nd | 1.80 | 0 | 2 | LFA-1 IP specific |
| 62 | IPI00216298 | TXN | thioredoxin | nd | 1.03 | 0 | 2 | LFA-1 IP specific |
| 63 | IPI00010779 | TPM4 | tropomyosin 4 | nd | 0.83 | 0 | 2 | LFA-1 IP specific |
| 64 | *IPI00025380* | *ITGAL* | *integrin, alpha L* | *0.02* | *175.91* | *1* | *41* | *9462.08* |
| 65 | IPI00550451 | PPP1CA | protein phosphatase 1, catalytic subunit, alpha isozyme | 35.74 | 89.63 | 11 | 17 | 2.51 |
| 66 | IPI00000874 | PRDX1 | peroxiredoxin 1 | 3.62 | 28.67 | 8 | 9 | 7.92 |
| 67 | IPI00217030 | RPS4X | ribosomal protein S4, X-linked | 2.49 | 13.16 | 3 | 9 | 5.28 |
| 68 | IPI00930688 | TUBA1B | tubulin, alpha 1b | 0.40 | 1.77 | 12 | 9 | 4.49 |
| 69 | IPI00005159 | ACTR2 | ARP2 actin-related protein 2 homolog (yeast) | 5.70 | 20.29 | 3 | 9 | 3.56 |
| 70 | IPI00306325 | PTPRC | protein tyrosine phosphatase, receptor type, C | 3.93 | 11.40 | 7 | 8 | 2.90 |
| 71 | IPI00221088 | RPS9 | ribosomal protein S9 | 0.57 | 7.65 | 1 | 7 | 13.35 |
| 72 | IPI00940386 | YTHDF3 | YTH domain family, member 3 | 0.31 | 1.80 | 2 | 7 | 5.74 |
| 73 | IPI00419585 | PPIA | peptidylprolyl isomerase A (cyclophilin A) | 3.28 | 10.76 | 2 | 7 | 3.28 |
| 74 | IPI00028091 | ACTR3 | ARP3 actin-related protein 3 homolog (yeast) | 0.54 | 1.61 | 8 | 7 | 2.99 |
| 75 | IPI00299573 | RPL7A | ribosomal protein L7a | 0.81 | 8.57 | 1 | 6 | 10.62 |
| 76 | IPI00419880 | RPS3A | ribosomal protein S3A | 0.65 | 4.39 | 1 | 6 | 6.75 |
| 77 | IPI00010896 | CLIC1 | chloride intracellular channel 1 | 0.45 | 1.67 | 1 | 6 | 3.73 |
| 78 | *IPI00219018* | *GAPDH* | *glyceraldehyde-3-phosphate dehydrogenase* | *3.72* | *12.49* | *3* | *6* | *3.35* |
| 79 | IPI00017376 | SEC23B | Sec23 homolog B (S. cerevisiae) | 0.62 | 1.54 | 4 | 6 | 2.50 |
| 80 | IPI00412579 | RPL10A | ribosomal protein L10a | 0.26 | 8.69 | 1 | 5 | 33.02 |
| 81 | IPI00218414 | CA2 | carbonic anhydrase II | 0.24 | 5.37 | 1 | 5 | 22.11 |
| 82 | *IPI00299571* | *PDIA6* | *protein disulfide isomerase family A, member 6* | *0.27* | *4.06* | *6* | *5* | *14.82* |
| 83 | IPI00926581 | MYH14 | myosin, heavy chain 14, non-muscle | 2.17 | 23.38 | 3 | 5 | 10.78 |
| 84 | IPI00221092 | RPS16 | ribosomal protein S16 | 1.76 | 13.90 | 2 | 5 | 7.88 |
| 85 | IPI00000494 | RPL5 | ribosomal protein L5 | 0.28 | 1.95 | 1 | 5 | 7.02 |
| 86 | IPI00306960 | NARS | asparaginyl-tRNA synthetase | 0.03 | 0.14 | 3 | 5 | 4.29 |
| 87 | IPI00024661 | SEC24C | SEC24 family member C | 0.10 | 0.31 | 2 | 5 | 3.25 |
| 88 | IPI00018235 | PEF1 | penta-EF-hand domain containing 1 | 0.54 | 1.72 | 5 | 5 | 3.20 |
| 89 | IPI00306933 | MYO9B | myosin IXB | 0.01 | 0.03 | 5 | 5 | 2.93 |
| 90 | IPI00012011 | CFL1 | cofilin 1 (non-muscle) | 0.67 | 8.05 | 1 | 4 | 12.09 |
| 91 | IPI00013485 | RPS2 | ribosomal protein S2 | 0.23 | 2.77 | 3 | 4 | 11.95 |
| 92 | IPI00003881 | HNRNPF | heterogeneous nuclear ribonucleoprotein F | 0.13 | 1.23 | 1 | 4 | 9.32 |
| 93 | IPI00304612 | RPL13A | ribosomal protein L13a | 0.51 | 3.82 | 2 | 4 | 7.47 |
| 94 | IPI00456969 | DYNC1H1 | dynein, cytoplasmic 1, heavy chain 1 | 0.01 | 0.03 | 1 | 4 | 5.37 |
| 95 | IPI00012772 | RPL8 | ribosomal protein L8 | 1.55 | 8.20 | 1 | 4 | 5.29 |
| 96 | IPI00479877 | ALDH9A1 | aldehyde dehydrogenase 9 family, member A1 | 0.49 | 2.41 | 1 | 4 | 4.89 |
| 97 | IPI00297779 | CCT2 | chaperonin containing TCP1, subunit 2 (beta) | 0.09 | 0.39 | 1 | 4 | 4.11 |
| 98 | *IPI00013933* | *DSP* | *desmoplakin* | *0.03* | *0.12* | *0* | *4* | *4.06* |
| 99 | IPI00644708 | TIAL1 | TIA1 cytotoxic granule-associated RNA binding protein-like 1 | 1.22 | 4.63 | 1 | 4 | 3.79 |
| 100 | IPI00221091 | RPS15A | ribosomal protein S15a | 2.42 | 8.48 | 1 | 41 | 3.51 |
| 101 | IPI00465361 | RPL13 | ribosomal protein L13 | 2.04 | 7.09 | 1 | 4 | 3.48 |
| 102 | IPI00873472 | SEC24A | SEC24 family member A | 0.02 | 0.07 | 2 | 4 | 3.48 |
| 103 | IPI00291006 | MDH2 | malate dehydrogenase 2, NAD (mitochondrial) | 0.61 | 1.95 | 5 | 4 | 3.21 |
| 104 | IPI00027107 | TUFM | Tu translation elongation factor, mitochondrial | 0.32 | 0.95 | 1 | 4 | 3.01 |
| 105 | IPI00183626 | PTBP1 | polypyrimidine tract binding protein 1 | 0.51 | 1.49 | 1 | 4 | 2.94 |
| 106 | IPI00003479 | MAPK1 | mitogen-activated protein kinase 1 | 0.64 | 1.82 | 2 | 4 | 2.85 |
| 107 | *IPI00293303* | *LGMN* | *legumain* | *0.06* | *1.13* | *1* | *4* | *19.53* |
| 108 | IPI00297492 | STT3A | STT3A, subunit of the oligosaccharyltransferase complex (catalytic) | 0.08 | 1.43 | 1 | 3 | 16.94 |
| 109 | IPI00024919 | PRDX3 | peroxiredoxin 3 | 0.42 | 4.01 | 3 | 3 | 9.47 |
| 110 | IPI00219155 | RPL27 | ribosomal protein L27 | 2.51 | 20.01 | 1 | 3 | 7.99 |
| 111 | IPI00219219 | LGALS1 | lectin, galactoside-binding, soluble, 1 | 0.54 | 4.17 | 1 | 3 | 7.79 |
| 112 | IPI00021263 | YWHAZ | tyrosine 3-monooxygenase/tryptophan 5-monooxygenase activation protein, zeta | 0.29 | 2.27 | 1 | 3 | 7.74 |
| 113 | IPI00871870 | ARPC3 | actin related protein 2/3 complex, subunit 3, 21kDa | 0.76 | 4.33 | 2 | 3 | 5.70 |
| 114 | IPI00216308 | VDAC1 | voltage-dependent anion channel 1 | 0.40 | 1.95 | 1 | 3 | 4.91 |
| 115 | IPI00011253 | RPS3 | ribosomal protein S3 | 1.70 | 7.94 | 2 | 3 | 4.68 |
| 116 | IPI00024933 | RPL12 | ribosomal protein L12 | 1.77 | 6.29 | 3 | 3 | 3.56 |
| 117 | IPI00215777 | SLC25A3 | solute carrier family 25 (mitochondrial carrier; phosphate carrier), member 3 | 0.17 | 0.60 | 1 | 3 | 3.47 |
| 118 | IPI00646240 | HIST2H2BF | histone cluster 2, H2bf | 1.17 | 3.84 | 1 | 3 | 3.29 |
| 119 | *IPI00646917* | *NUDT21* | *nudix (nucleoside diphosphate linked moiety X)-type motif 21* | *1.17* | *3.84* | *1* | *3* | *3.29* |
| 120 | IPI00218187 | PPP1CC | protein phosphatase 1, catalytic subunit, gamma isozyme | 1.15 | 3.66 | 1 | 3 | 3.19 |
| 121 | IPI00010415 | ACOT7 | acyl-CoA thioesterase 7 | 0.38 | 1.17 | 0 | 3 | 3.12 |
| 122 | IPI00023598 | TUBB4A | tubulin, beta 4A class IVa | 0.41 | 1.25 | 1 | 3 | 3.03 |
| 123 | IPI00884105 | LAMP1 | lysosomal-associated membrane protein 1 | 0.28 | 0.83 | 2 | 3 | 2.97 |
| 124 | IPI00221089 | RPS13 | ribosomal protein S13 | 1.12 | 3.28 | 1 | 3 | 2.93 |
| 125 | IPI00008438 | RPS10 | ribosomal protein S10 | 1.24 | 3.58 | 2 | 3 | 2.89 |
| 126 | IPI00305305 | SIPA1 | signal-induced proliferation-associated 1 | 0.15 | 0.41 | 6 | 3 | 2.84 |
| 127 | IPI00001883 | SNX9 | sorting nexin 9 | 0.61 | 1.60 | 2 | 3 | 2.61 |
| 128 | IPI00016610 | PCBP1 | poly(rC) binding protein 1 | 1.10 | 2.78 | 5 | 3 | 2.51 |
| 129 | IPI00006091 | DMD | dystrophin | 0.10 | 1.25 | 4 | 3 | 12.62 |
| 130 | IPI00376798 | RPL11 | ribosomal protein L11 | 1.27 | 13.40 | 1 | 2 | 10.55 |
| 131 | IPI00008433 | RPS5 | ribosomal protein S5 | 0.18 | 1.77 | 1 | 2 | 9.73 |
| 132 | IPI00639957 | CARM1 | coactivator-associated arginine methyltransferase 1 | 0.05 | 0.43 | 0 | 2 | 9.26 |
| 133 | IPI00026302 | RPL31 | ribosomal protein L31 | 0.76 | 5.35 | 3 | 2 | 7.08 |
| 134 | IPI00470528 | RPL15 | ribosomal protein L15 | 0.37 | 2.63 | 2 | 2 | 7.03 |
| 135 | IPI00215918 | ARF4 | ADP-ribosylation factor 4 | 0.11 | 0.62 | 2 | 2 | 5.49 |
| 136 | IPI00017448 | RPS21 | ribosomal protein S21 | 1.15 | 6.28 | 1 | 2 | 5.48 |
| 137 | IPI00010402 | SH3BGRL3 | SH3 domain binding glutamate-rich protein like 3 | 2.47 | 10.53 | 1 | 2 | 4.26 |
| 138 | IPI00021428 | ACTA1 | actin, alpha 1, skeletal muscle | 10.27 | 38.19 | 1 | 2 | 3.72 |
| 139 | IPI00012585 | HEXB | hexosaminidase B (beta polypeptide) | 0.14 | 0.49 | 2 | 2 | 3.42 |
| 140 | IPI00455134 | HNRNPA3 | heterogeneous nuclear ribonucleoprotein A3 | 0.74 | 2.10 | 1 | 2 | 2.82 |
| 141 | IPI00016249 | FXR1 | fragile X mental retardation, autosomal homolog 1 | 0.10 | 0.28 | 1 | 2 | 2.70 |
| 142 | IPI00021828 | CSTB | cystatin B (stefin B) | 13.27 | 34.97 | 4 | 2 | 2.63 |
| 143 | IPI00023530 | CDK5 | cyclin-dependent kinase 5 | 0.07 | 0.17 | 3 | 2 | 2.56 |
| 144 | IPI00099463 | SGPL1 | sphingosine-1-phosphate lyase 1 | 0.10 | 0.25 | 1 | 2 | 2.52 |
| 145 | *IPI00029741* | *ITGB5* | *integrin, beta 5* | *0.14* | *14.03* | *1* | *2* | *97.15* |
| 146 | IPI00026202 | RPL18A | ribosomal protein L18a | 0.48 | 10.12 | 1 | 1 | 21.22 |
| 147 | IPI00555744 | RPL14 | ribosomal protein L14 | 1.45 | 10.71 | 1 | 1 | 7.41 |
| 148 | IPI00029012 | EIF3A | eukaryotic translation initiation factor 3, subunit A | 0.05 | 0.24 | 1 | 1 | 5.22 |
| 149 | IPI00219153 | RPL22 | ribosomal protein L22 | 1.99 | 8.89 | 0 | 1 | 4.46 |
| 150 | IPI00017375 | SEC23A | Sec23 homolog A (S. cerevisiae) | 0.07 | 0.28 | 1 | 1 | 3.89 |
| 151 | IPI00556231 | LOC644936 | actin, beta pseudogene | 14.53 | 55.26 | 1 | 1 | 3.80 |
| 152 | IPI00007853 | IFI30 | interferon, gamma-inducible protein 30 | 0.95 | 3.44 | 1 | 1 | 3.61 |
| 153 | IPI00947127 | LDHA | lactate dehydrogenase A | 0.07 | 0.23 | 1 | 1 | 3.21 |
| 154 | IPI00024742 | UQCRQ | ubiquinol-cytochrome c reductase, complex III subunit VII, 9.5kDa | 1.39 | 4.27 | 1 | 1 | 3.07 |
| 155 | IPI00472523 | LGALS9B | lectin, galactoside-binding, soluble, 9B | 0.65 | 1.73 | 1 | 1 | 2.67 |
| 156 | IPI00945633 | SSR1 | signal sequence receptor, alpha | 0.12 | 0.31 | 1 | 1 | 2.64 |
| 157 | IPI00001885 | SNX8 | sorting nexin 8 | 0.10 | 0.26 | 1 | 1 | 2.55 |
| 158 | IPI00005969 | CAPZA1 | capping protein (actin filament) muscle Z-line, alpha 1 | 0.10 | 0.26 | 2 | 1 | 2.55 |
| 159 | IPI00293276 | MIF | macrophage migration inhibitory factor (glycosylation-inhibiting factor) | 0.37 | 1.18 | 2 | 1 | 3.22 |
| 160 | IPI00940084 | TPM1 | tropomyosin 1 (alpha) | 0.04 | 0.10 | 1 | 0 | 2.60 |
| 161 | IPI00376344 | MYO1B | myosin IB | 0.03 | 0.08 | 0 | 0 | 2.58 |
