# Supplementary material for: Proteome Based Construction of the Lymphocyte Function-Associated Antigen 1 (LFA-1) Interactome in Human Dendritic Cells
Source: PLoS One. 2016 Feb 18;11(2):e0149637. doi: 10.1371/journal.pone.0149637 (PMC4758637; doi:10.1371/journal.pone.0149637)
Supplement: S2 Table — The criteria for protein selection were the following: proteins were at least 2.5 fold enriched over the isotype-type matched control IP (based on the normalized IBAQ value), or were specifically detected in the LFA-1 IP with a minimum of 2 and 3 unique peptides in mild and stringent IP conditions, respectively. Proteins were mapped to HGNC and International Protein Index (IPI) identifiers. Italic indicates proteins detected in multiple experiments and IBAQ values and peptide counts were taken from the best experiment. (DOCX) [file pone.0149637.s002.docx]

**S2 Table:**

|  | **Mild IP condition**  **Protein AcNR** | **HGNC symbol** | **Protein Name (IPA)** | **normalized IBAQ control IP** | **normalized IBAQ LFA-1 IP** | **max peptides control** | **max peptides LFA-1 IP** | **fold change (LFA-1 IP/Control IP)** |
| --- | --- | --- | --- | --- | --- | --- | --- | --- |
| 1 | *IPI00291792* | *ITGB2* | *integrin, beta 2* | *0* | *524.87* | *0* | *35* | *LFA-1 IP specific* |
| 2 | IPI00013877 | HNRNPH3 | heterogeneous nuclear ribonucleoprotein H3 (2H9) | 0 | 3.24 | 0 | 5 | LFA-1 IP specific |
| 3 | IPI00328257 | AP1B1 | adaptor-related protein complex 1, beta 1 subunit | 0 | 1.54 | 0 | 5 | LFA-1 IP specific |
| 4 | *IPI00465248* | *ENO1* | *enolase 1, (alpha)* | *0* | *2.67* | *0* | *5* | *LFA-1 IP specific* |
| 5 | IPI00646773 | GSN | gelsolin | 0 | 1.14 | 0 | 5 | LFA-1 IP specific |
| 6 | IPI00215965 | HNRNPA1 | heterogeneous nuclear ribonucleoprotein A1 | 0 | 1.34 | 0 | 4 | LFA-1 IP specific |
| 7 | IPI00305383 | UQCRC2 | ubiquinol-cytochrome c reductase core protein II | 0 | 2.88 | 0 | 4 | LFA-1 IP specific |
| 8 | IPI00947127 | LDHA | lactate dehydrogenase A | 0 | 4.73 | 0 | 4 | LFA-1 IP specific |
| 9 | IPI00023020 | SEMG1 | semenogelin I | 0 | 1.05 | 0 | 3 | LFA-1 IP specific |
| 10 | IPI00260715 | FUS | FUS RNA binding protein | 0 | 2.62 | 0 | 3 | LFA-1 IP specific |
| 11 | IPI00299571 | PDIA6 | protein disulfide isomerase family A, member 6 | 0 | 3.79 | 0 | 3 | LFA-1 IP specific |
| 12 | IPI00337494 | SLC25A24 | solute carrier family 25 (mitochondrial carrier; phosphate carrier), member 24 | 0 | 0.71 | 0 | 3 | LFA-1 IP specific |
| 13 | IPI00645948 | HMGB1 | high mobility group box 1 | 0 | 4.16 | 0 | 3 | LFA-1 IP specific |
| 14 | IPI00006091 | DMD | dystrophin | 0 | 4.31 | 0 | 2 | LFA-1 IP specific |
| 15 | IPI00009342 | IQGAP1 | IQ motif containing GTPase activating protein 1 | 0 | 0.08 | 0 | 2 | LFA-1 IP specific |
| 16 | IPI00010270 | RAC2 | ras-related C3 botulinum toxin substrate 2 (rho family, small GTP binding protein Rac2) | 0 | 1.88 | 0 | 2 | LFA-1 IP specific |
| 17 | IPI00013070 | HNRNPUL1 | heterogeneous nuclear ribonucleoprotein U-like 1 | 0 | 0.19 | 0 | 2 | LFA-1 IP specific |
| 18 | IPI00019755 | GSTO1 | glutathione S-transferase omega 1 | 0 | 0.90 | 0 | 2 | LFA-1 IP specific |
| 19 | IPI00024067 | CLTC | clathrin, heavy chain (Hc) | 0 | 0.13 | 0 | 2 | LFA-1 IP specific |
| 20 | IPI00027230 | HSP90B1 | heat shock protein 90kDa beta (Grp94), member 1 | 0 | 0.13 | 0 | 2 | LFA-1 IP specific |
| 21 | IPI00028888 | HNRNPD | heterogeneous nuclear ribonucleoprotein D (AU-rich element RNA binding protein 1, 37kDa) | 0 | 1.45 | 0 | 2 | LFA-1 IP specific |
| 22 | IPI00152377 | STT3B | STT3B, subunit of the oligosaccharyltransferase complex (catalytic) | 0 | 0.24 | 0 | 2 | LFA-1 IP specific |
| 23 | IPI00186290 | EEF2 | eukaryotic translation elongation factor 2 | 0 | 0.26 | 0 | 2 | LFA-1 IP specific |
| 24 | IPI00215637 | DDX3X | DEAD (Asp-Glu-Ala-Asp) box helicase 3, X-linked | 0 | 0.14 | 0 | 2 | LFA-1 IP specific |
| 25 | IPI00218918 | ANXA1 | annexin A1 | 0 | 0.38 | 0 | 2 | LFA-1 IP specific |
| 26 | IPI00219153 | RPL22 | ribosomal protein L22 | 0 | 5.36 | 0 | 2 | LFA-1 IP specific |
| 27 | IPI00219219 | LGALS1 | lectin, galactoside-binding, soluble, 1 | 0 | 1.89 | 0 | 2 | LFA-1 IP specific |
| 28 | IPI00219729 | SLC25A11 | solute carrier family 25 (mitochondrial carrier; oxoglutarate carrier), member 11 | 0 | 0.63 | 0 | 2 | LFA-1 IP specific |
| 29 | IPI00220300 | ATP5J2 | ATP synthase, H+ transporting, mitochondrial Fo complex, subunit F2 | 0 | 3.35 | 0 | 2 | LFA-1 IP specific |
| 30 | IPI00296099 | THBS1 | thrombospondin 1 | 0 | 0.17 | 0 | 2 | LFA-1 IP specific |
| 31 | IPI00304742 | STK10 | serine/threonine kinase 10 | 0 | 0.60 | 0 | 2 | LFA-1 IP specific |
| 32 | IPI00334587 | HNRNPAB | heterogeneous nuclear ribonucleoprotein A/B | 0 | 1.41 | 0 | 2 | LFA-1 IP specific |
| 33 | IPI00337541 | NNT | nicotinamide nucleotide transhydrogenase | 0 | 0.23 | 0 | 2 | LFA-1 IP specific |
| 34 | IPI00455134 | HNRNPA3 | heterogeneous nuclear ribonucleoprotein A3 | 0 | 3.00 | 0 | 2 | LFA-1 IP specific |
| 35 | IPI00642816 | SRP9 | signal recognition particle 9kDa | 0 | 1.25 | 0 | 2 | LFA-1 IP specific |
| 36 | IPI00797126 | NACA | nascent polypeptide-associated complex alpha subunit | 0 | 0.66 | 0 | 2 | LFA-1 IP specific |
| 37 | IPI00873935 | DKC1 | dyskeratosis congenita 1, dyskerin | 0 | 0.45 | 0 | 2 | LFA-1 IP specific |
| 38 | IPI00909772 | AP2M1 | adaptor-related protein complex 2, mu 1 subunit | 0 | 1.73 | 0 | 2 | LFA-1 IP specific |
| 39 | IPI00789324 | JUP | junction plakoglobin | 0 | 0.22 | 0 | 0 | LFA-1 IP specific |
| 40 | *IPI00025380* | *ITGAL* | *integrin, alpha L* | *0.11* | *352.80* | *0* | *40* | *3096.53* |
| 41 | IPI00396378 | HNRNPA2B1 | heterogeneous nuclear ribonucleoprotein A2/B1 | 0.12 | 52.50 | 1 | 17 | 421.84 |
| 42 | IPI00296713 | GRN | granulin | 0.30 | 9.93 | 1 | 9 | 32.70 |
| 43 | IPI00168728 | IGHM | immunoglobulin heavy constant mu | 0.37 | 10.23 | 1 | 2 | 27.72 |
| 44 | IPI00013847 | UQCRC1 | ubiquinol-cytochrome c reductase core protein I | 0.10 | 1.98 | 1 | 3 | 20.76 |
| 45 | IPI00003269 | ACTBL2 | actin, beta-like 2 | 22.62 | 271.32 | 1 | 2 | 12.00 |
| 46 | IPI00010341 | PRG2 | proteoglycan 2, bone marrow (natural killer cell activator, eosinophil granule major basic protein) | 0.21 | 1.95 | 0 | 3 | 9.45 |
| 47 | IPI00011229 | CTSD | cathepsin D | 1.12 | 9.95 | 3 | 2 | 8.90 |
| 48 | IPI00872350 | GALC | galactosylceramidase | 0.31 | 2.48 | 1 | 5 | 7.92 |
| 49 | IPI00930688 | TUBA1B | tubulin, alpha 1b | 0.07 | 0.54 | 1 | 2 | 7.42 |
| 50 | IPI00296909 | PARP4 | poly (ADP-ribose) polymerase family, member 4 | 0.16 | 1.10 | 3 | 9 | 6.77 |
| 51 | IPI00003362 | HSPA5 | heat shock 70kDa protein 5 (glucose-regulated protein, 78kDa) | 1.02 | 6.71 | 7 | 13 | 6.57 |
| 52 | IPI00007611 | ATP5O | ATP synthase, H+ transporting, mitochondrial F1 complex, O subunit | 0.41 | 2.55 | 1 | 5 | 6.27 |
| 53 | IPI00221089 | RPS13 | ribosomal protein S13 | 1.30 | 8.07 | 1 | 2 | 6.21 |
| 54 | IPI00291006 | MDH2 | malate dehydrogenase 2, NAD (mitochondrial) | 0.21 | 1.28 | 1 | 4 | 5.99 |
| 55 | IPI00398779 | PLEC | plectin | 0.17 | 1.00 | 2 | 1 | 5.92 |
| 56 | IPI00011285 | CAPN1 | calpain 1, (mu/I) large subunit | 0.05 | 0.32 | 1 | 1 | 5.91 |
| 57 | IPI00027252 | PHB2 | prohibitin 2 | 0.85 | 4.98 | 3 | 7 | 5.86 |
| 58 | IPI00299150 | CTSS | cathepsin S | 2.28 | 13.31 | 3 | 5 | 5.85 |
| 59 | IPI00019345 | RAP1A | RAP1A, member of RAS oncogene family | 0.45 | 2.44 | 1 | 2 | 5.49 |
| 60 | IPI00383581 | GANAB | glucosidase, alpha; neutral AB | 0.08 | 0.44 | 1 | 1 | 5.47 |
| 61 | IPI00021711 | HLA-DPA1 | major histocompatibility complex, class II, DP alpha 1 | 0.34 | 1.80 | 1 | 1 | 5.31 |
| 62 | IPI00218646 | CYBB | cytochrome b-245, beta polypeptide | 0.12 | 0.61 | 1 | 3 | 5.07 |
| 63 | IPI00179330 | RPS27A | ribosomal protein S27a | 0.90 | 4.45 | 1 | 2 | 4.92 |
| 64 | IPI00026202 | RPL18A | ribosomal protein L18a | 0.91 | 4.35 | 1 | 4 | 4.79 |
| 65 | IPI00215884 | SRSF1 | serine/arginine-rich splicing factor 1 | 0.28 | 1.32 | 0 | 2 | 4.70 |
| 66 | IPI00219365 | MSN | moesin | 0.11 | 0.49 | 1 | 3 | 4.41 |
| 67 | IPI00470528 | RPL15 | ribosomal protein L15 | 0.31 | 1.33 | 1 | 2 | 4.26 |
| 68 | IPI00012555 | FCN1 | ficolin (collagen/fibrinogen domain containing) 1 | 2.16 | 9.17 | 2 | 4 | 4.26 |
| 69 | IPI00019038 | LYZ | lysozyme | 55.81 | 225.54 | 4 | 7 | 4.04 |
| 70 | IPI00017510 | MT-CO2 | cytochrome c oxidase subunit II | 3.54 | 14.02 | 1 | 4 | 3.97 |
| 71 | IPI00007750 | TUBA4A | tubulin, alpha 4a | 0.23 | 0.89 | 1 | 2 | 3.90 |
| 72 | IPI00304612 | RPL13A | ribosomal protein L13a | 0.18 | 0.68 | 1 | 1 | 3.87 |
| 73 | IPI00479186 | PKM | pyruvate kinase, muscle | 1.80 | 6.75 | 6 | 14 | 3.75 |
| 74 | IPI00019563 | GIMAP4 | GTPase, IMAP family member 4 | 0.24 | 0.89 | 1 | 3 | 3.72 |
| 75 | IPI00420108 | ANXA2 | annexin A2 | 2.10 | 7.73 | 2 | 3 | 3.69 |
| 76 | IPI00022446 | PF4 | platelet factor 4 | 5.09 | 18.29 | 2 | 2 | 3.59 |
| 77 | IPI00013163 | MNDA | myeloid cell nuclear differentiation antigen | 5.24 | 18.49 | 5 | 7 | 3.53 |
| 78 | IPI00641737 | NDUFS1 | NADH dehydrogenase (ubiquinone) Fe-S protein 1, 75kDa (NADH-coenzyme Q reductase) | 2.88 | 10.15 | 4 | 8 | 3.52 |
| 79 | IPI00646304 | PPIB | peptidylprolyl isomerase B (cyclophilin B) | 0.21 | 0.74 | 1 | 2 | 3.51 |
| 80 | IPI00414676 | HSP90AB1 | heat shock protein 90kDa alpha (cytosolic), class B member 1 | 0.24 | 0.85 | 2 | 2 | 3.50 |
| 81 | IPI00328296 | GIPC2 | GIPC PDZ domain containing family, member 2 | 0.12 | 0.42 | 1 | 1 | 3.45 |
| 82 | IPI00018971 | TRIM21 | tripartite motif containing 21 | 14.41 | 49.52 | 12 | 18 | 3.44 |
| 83 | IPI00216730 | HIST2H2AB | histone cluster 2, H2ab | 1.63 | 5.55 | 1 | 1 | 3.41 |
| 84 | IPI00411680 | PCMT1 | protein-L-isoaspartate (D-aspartate) O-methyltransferase | 0.20 | 0.66 | 1 | 1 | 3.35 |
| 85 | IPI00013508 | ACTN1 | actinin, alpha 1 | 0.06 | 0.21 | 2 | 3 | 3.30 |
| 86 | IPI00654820 | MT-ATP6 | ATP synthase F0 subunit 6 | 0.95 | 2.97 | 1 | 1 | 3.14 |
| 87 | *IPI00440493* | *ATP5A1* | *ATP synthase, H+ transporting, mitochondrial F1 complex, alpha subunit 1, cardiac muscle* | *0.23* | *0.71* | *1* | *2* | *3.13* |
| 88 | IPI00303476 | ATP5B | ATP synthase, H+ transporting, mitochondrial F1 complex, beta polypeptide | 1.99 | 6.17 | 6 | 7 | 3.10 |
| 89 | IPI00217030 | RPS4X | ribosomal protein S4, X-linked | 0.10 | 0.31 | 1 | 1 | 3.05 |
| 90 | IPI00010896 | CLIC1 | chloride intracellular channel 1 | 0.16 | 0.47 | 1 | 2 | 3.04 |
| 91 | IPI00412579 | RPL10A | ribosomal protein L10a | 0.26 | 0.78 | 1 | 1 | 2.97 |
| 92 | IPI00003919 | QPCT | glutaminyl-peptide cyclotransferase | 0.68 | 1.99 | 1 | 3 | 2.94 |
| 93 | IPI00013933 | DSP | desmoplakin | 0.16 | 0.48 | 4 | 11 | 2.92 |
| 94 | IPI00784865 | IGK | immunoglobulin kappa locus | 1.00 | 2.85 | 1 | 1 | 2.85 |
| 95 | IPI00025252 | PDIA3 | protein disulfide isomerase family A, member 3 | 0.06 | 0.16 | 0 | 1 | 2.84 |
| 96 | IPI00294739 | SAMHD1 | SAM domain and HD domain 1 | 0.36 | 1.01 | 3 | 2 | 2.84 |
| 97 | IPI00221091 | RPS15A | ribosomal protein S15a | 4.24 | 11.91 | 3 | 5 | 2.81 |
| 98 | IPI00005724 | LANCL1 | LanC lantibiotic synthetase component C-like 1 (bacterial) | 0.09 | 0.26 | 1 | 1 | 2.79 |
| 99 | *IPI00796333* | *ALDOA* | *aldolase A, fructose-bisphosphate* | *1.17* | *3.19* | *3* | *3* | *2.72* |
| 100 | IPI00011253 | RPS3 | ribosomal protein S3 | 0.69 | 1.88 | 1 | 1 | 2.71 |
| 101 | IPI00003865 | HSPA8 | heat shock 70kDa protein 8 | 3.99 | 10.50 | 12 | 15 | 2.63 |
| 102 | IPI00926935 | GNAI2 | guanine nucleotide binding protein (G protein), alpha inhibiting activity polypeptide 2 | 1.61 | 4.22 | 3 | 3 | 2.62 |
| 103 | IPI00399007 | IGHG2 | immunoglobulin heavy constant gamma 2 (G2m marker) | 0.58 | 1.49 | 1 | 1 | 2.57 |
| 104 | IPI00006579 | COX4I1 | cytochrome c oxidase subunit IV isoform 1 | 2.08 | 5.34 | 2 | 2 | 2.57 |
| 105 | IPI00236556 | MPO | myeloperoxidase | 4.61 | 11.79 | 10 | 13 | 2.56 |
| 106 | IPI00027409 | PRTN3 | proteinase 3 | 4.44 | 11.34 | 2 | 2 | 2.56 |
| 107 | IPI00027462 | S100A9 | S100 calcium binding protein A9 | 1.78 | 4.48 | 1 | 3 | 2.51 |
| 108 | IPI00939667 | TPP1 | tripeptidyl peptidase I | 0.85 | 2.14 | 1 | 4 | 2.50 |
|  |  |  |  |  |  |  |  |  |
|  |  |  |  |  |  |  |  |  |
|  | **Stringent IP conditions**  **Protein**  **AcNR** | **HGNC**  **symbol** | **Protein Name (IPA)** | **normalized IBAQ control IP** | **normalized IBAQ LFA-1 IP** | **max peptides control** | **max peptides LFA-1 IP** | **fold change (LFA-1 IP/Control IP)** |
| 1 | IPI00909703 | ANXA11 | annexin A11 | 0 | 5 | 0 | 2.49 | LFA-1 IP specific |
| 2 | IPI00010270 | RAC2 | ras-related C3 botulinum toxin substrate 2 (rho family, small GTP binding protein Rac2) | 0 | 3 | 0 | 1 | LFA-1 IP specific |
| 3 | IPI00218918 | ANXA1 | annexin A1 | 0 | 3 | 0 | 0.54 | LFA-1 IP specific |
| 4 | IPI00219219 | LGALS1 | lectin, galactoside-binding, soluble, 1 | 0 | 3 | 0 | 3.61 | LFA-1 IP specific |
| 5 | IPI00291792 | ITGB2 | integrin, beta 2 (complement component 3 receptor 3 and 4 subunit) | 1 | 33 | 0.17 | 264.80 | 1514.47 |
| 6 | IPI00221226 | ANXA6 | annexin A6 | 11 | 22 | 2.49 | 26.49 | 10.65 |
| 7 | IPI00793199 | ANXA4 | annexin A4 | 1 | 12 | 0.12 | 20.25 | 172.53 |
| 8 | IPI00002460 | ANXA7 | annexin A7 | 1 | 9 | 0.14 | 5.25 | 36.57 |
| 9 | IPI00329801 | ANXA5 | annexin A5 | 3 | 8 | 0.43 | 6.83 | 15.94 |
| 10 | IPI00654755 | HBB | hemoglobin, beta | 2 | 4 | 2.37 | 9.86 | 4.15 |
| 11 | IPI00219018 | GAPDH | glyceraldehyde-3-phosphate dehydrogenase | 3 | 4 | 1.81 | 5.02 | 2.77 |
| 12 | IPI00215914 | ARF1 | ADP-ribosylation factor 1 | 1 | 3 | 0.54 | 1.55 | 2.86 |
| 13 | IPI00304925 | STK10 | serine/threonine kinase 10 | 1 | 3 | 0.07 | 0.17 | 2.50 |
| 14 | IPI00003269 | ACTBL2 | actin, beta-like 2 | 1 | 1 | 0.95 | 93.84 | 98.43 |
| 15 | IPI00410714 | IGHG2 | immunoglobulin heavy constant gamma 2 (G2m marker) | 1 | 1 | 0.38 | 1.87 | 4.93 |
| 16 | IPI00472523 | LGALS9B | lectin, galactoside-binding, soluble, 9B | 1 | 1 | 12.96 | 35.57 | 2.74 |
